# Rapid rise in premature mortality due to anthropogenic air pollution in fast-growing tropical cities from 2005 to 2018

**DOI:** 10.1126/sciadv.abm4435

**Published:** 2022-04-08

**Authors:** Karn Vohra, Eloise A. Marais, William J. Bloss, Joel Schwartz, Loretta J. Mickley, Martin Van Damme, Lieven Clarisse, Pierre-F. Coheur

**Affiliations:** 1 School of Geography, Earth, and Environmental Sciences, University of Birmingham, Birmingham, UK.; 2 Department of Geography, University College London, London, UK.; 3 Department of Environmental Health, Harvard T.H. Chan School of Public Health, Harvard University, Boston, MA, USA.; 4 John A. Paulson School of Engineering and Applied Sciences, Harvard University, Cambridge, MA, USA.; 5 Université libre de Bruxelles (ULB), Spectroscopy, Quantum Chemistry and Atmospheric Remote Sensing (SQUARES), Brussels, Belgium.

## Abstract

Tropical cities are experiencing rapid growth but lack routine air pollution monitoring to develop prescient air quality policies. Here, we conduct targeted sampling of recent (2000s to 2010s) observations of air pollutants from space-based instruments over 46 fast-growing tropical cities. We quantify significant annual increases in nitrogen dioxide (NO_2_) (1 to 14%), ammonia (2 to 12%), and reactive volatile organic compounds (1 to 11%) in most cities, driven almost exclusively by emerging anthropogenic sources rather than traditional biomass burning. We estimate annual increases in urban population exposure to air pollutants of 1 to 18% for fine particles (PM_2.5_) and 2 to 23% for NO_2_ from 2005 to 2018 and attribute 180,000 (95% confidence interval: −230,000 to 590,000) additional premature deaths in 2018 (62% increase relative to 2005) to this increase in exposure. These cities are predicted to reach populations of up to 80 million people by 2100, so regulatory action targeting emerging anthropogenic sources is urgently needed.

## Teaser

A total of 180,000 additional premature deaths are attributable to rapid air quality degradation in fast-growing tropical cities.

## INTRODUCTION

More than 40% of the global population resides in the tropics (*1*). Of those, less than half reside in urban areas, although this is expected to exceed 50% by 2050 because of steep rates of urbanization and unprecedented population growth (*1*). By 2100, most (51 of 70) global megacities are projected to be in the tropics (25°S to 25°N), concentrated in Africa and Asia (*2*). This has the potential for severe impacts on air quality and climate, as megacities represent an overwhelming contribution to carbon and air pollutant emissions (*3*, *4*). Many countries in the tropics have yet to implement robust policies and necessary infrastructure to monitor and mitigate air pollution (*5*). Even where policies exist, such as in large cities in India, there is limited evidence of remediation (*6*). Knowledge of recent trends in the sources, abundance, and population exposure to air pollution in these rapidly growing cities is crucial for demonstrating the scale of air quality degradation and to hasten adoption of sustainable mitigation measures to avoid repeating past air pollution health crises.

Ambient air pollution in much of the tropics is dominated by widespread, intense seasonal open burning of biomass (*7*, *8*). The contribution from anthropogenic activity varies regionally and is greatest in tropical Asia, predominantly from residential combustion and industrial activity (*7*, *9*). Air pollution in tropical Africa is also influenced by natural sources (desert dust and biogenic emissions) as well as residential and commercial production and use of solid fuels (*10*–*12*). Seasonality in meteorology affects air quality, particularly in West Africa (*13*) and India (*14*), where southwesterly winds and heavy rainfall during the monsoon season disperse and wash away pollution and stagnant conditions during the dry season lead to its accumulation. Combined rapid growth in anthropogenic activity and very efficient tropical deep convective injection of pollutants and precursors to the free troposphere also have the potential to greatly influence global atmospheric chemistry and climate (*15*). The relative role of natural, anthropogenic, and biomass burning sources to trends in air pollution in the tropics remains elusive (*7*, *16*, *17*).

Exposure to ambient fine particulate pollution (PM_2.5_) is already a leading environmental health risk in many countries in the tropics (*5*, *18*). More than 30% of premature deaths in Asia are attributable to exposure to PM_2.5_ from fossil fuel combustion alone (*19*), and 170,000 global premature infant deaths, mostly in South Asia and sub-Saharan Africa, have been attributed to exposure to PM_2.5_ (*20*). Annual mean population-weighted PM_2.5_ concentrations for the tropics are almost five times the recently updated World Health Organization (WHO) guideline of 5 μg m^−3^ (*21*), although this was determined with very few measurements (<1 monitor per million people in many tropical countries) (*22*). Monitoring capacity has improved with deployment of low-cost sensors and additional reference-grade instruments (*23*, *24*), but large data gaps as well as data quality and access issues remain. India, for example, has an extensive network of monitors operated and maintained by local and national authorities as well as research institutions, but the use of these for informing policies is hindered by data quality issues for the national network (*6*, *23*) and restricted access to data collected by research institutions and state governments.

Satellite observations provide long-term, consistent, global observations of a range of chemical components of the atmosphere. These offer constraints on long-term changes in abundance of surface air pollutants, precursor emissions of short-lived air pollutants (*25*, *26*), and sensitivity of ozone formation to source types for informing policy measures to regulate already severe ozone pollution in the tropics (*8*). Past studies have typically focused on a single pollutant observable from space-based sensors over current megacities, mostly in the northern hemisphere (*27*–*29*). Many fast-growing tropical cities are anticipated to reach 40 million to 80 million inhabitants by 2100 (*2*), far greater than the 10 largest contemporary megacities of 20 million to 40 million (*1*). In our previous work, we demonstrated that space-based observations of integrated columns of nitrogen dioxide (NO_2_) and ammonia (NH_3_) abundances as well as aerosol optical depth (AOD) reproduce long-term trends in surface observations of NO_2_, NH_3_, and PM_2.5_, respectively (*6*). Column abundances of formaldehyde (HCHO), a ubiquitous oxidation product of volatile organic compounds (VOCs), provide constraints on reactive VOCs (*30*).

Here, we conduct targeted sampling of more than a decade of satellite observations of NO_2_, NH_3_, AOD, and HCHO over fast-growing tropical cities to determine recent (2000s to 2010s) trends in air pollution abundance and precursor emissions and to discern the relative role of traditional and emerging pollution sources. We go on to estimate the increase in urban population exposure to the hazardous pollutants PM_2.5_ and NO_2_ and the associated health burden of exposure to PM_2.5_ using a health risk assessment model constrained with epidemiological data representative of the range of PM_2.5_ concentrations in the tropics.

## RESULTS

### Trends in air quality in fast-growing tropical cities

**Fig. 1.**
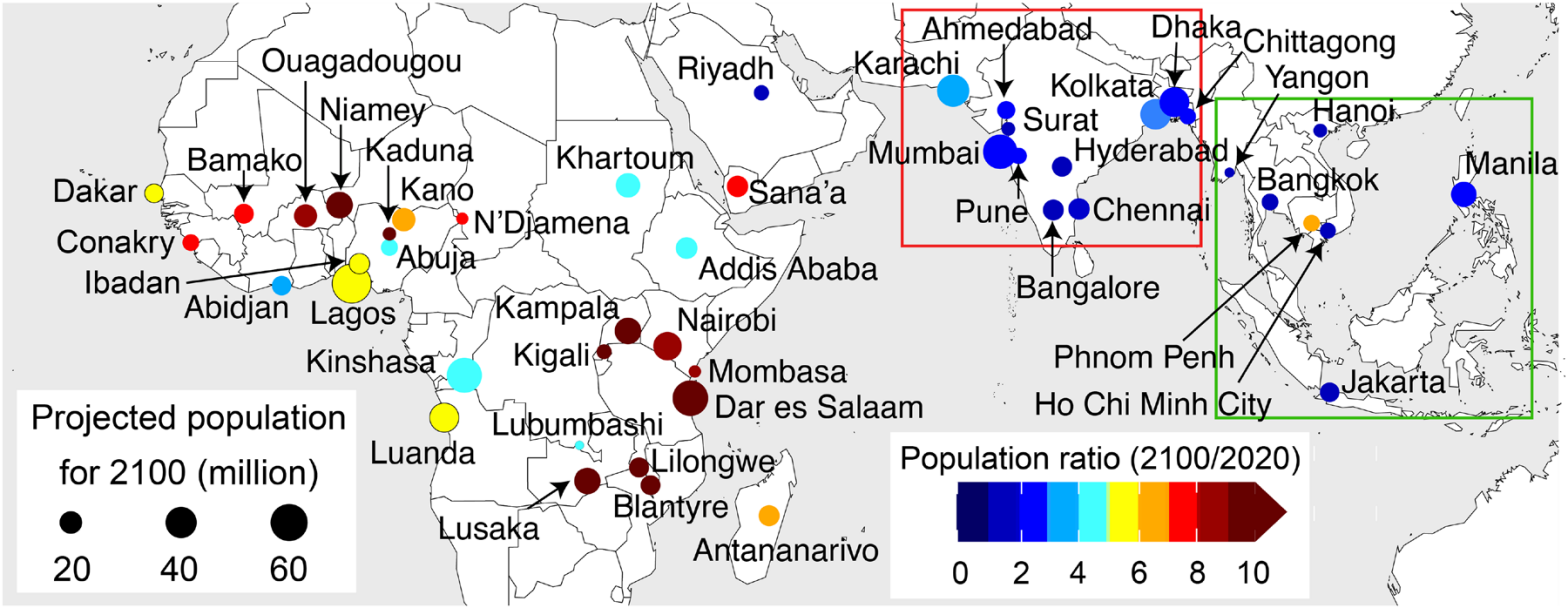
Projected population growth for the fastest-growing cities in the tropics (25°S to 25°N) anticipated to be megacities (population ≥ 10 million) by 2100. Circle sizes indicate the projected 2100 population, and colors are 2100-to-2020 population ratios as indicators of population growth. Data for 2100 are from Hoornweg and Pope (*2*) and, for 2020, from the United Nations (UN) (*1*). Boxes discern cities in South Asia (red) and Southeast Asia (green).

The 46 cities in tropical Africa, the Middle East, and Asia that are projected to be megacities (population ≥ 10 million) by 2100 are shown in Fig. 1. Only 12 are megacities now, mostly in India. Forecast population growth rates from 2020 to 2100 range from 3 to 31% per year (a^−1^) in Africa, 1% a^−1^ for Riyadh and 8% a^−1^ for Sana’a in the Middle East, 0.8 to 3% a^−1^ in South Asia, and 0.5 to 7% a^−1^ in Southeast Asia (*2*). The largest cities, predicted to surpass 50 million inhabitants by 2100, include Lagos (80 million) in Nigeria, Dar es Salaam (62 million) in Tanzania, Kinshasa (60 million) in the Democratic Republic of the Congo, and Mumbai (58 million) in India. There are also five cities in tropical America that are already megacities (fig. S1), but our focus is on the cluster of tropical cities in Asia, Africa, and the Middle East because of their much faster projected growth (*2*).

**Fig. 2.**
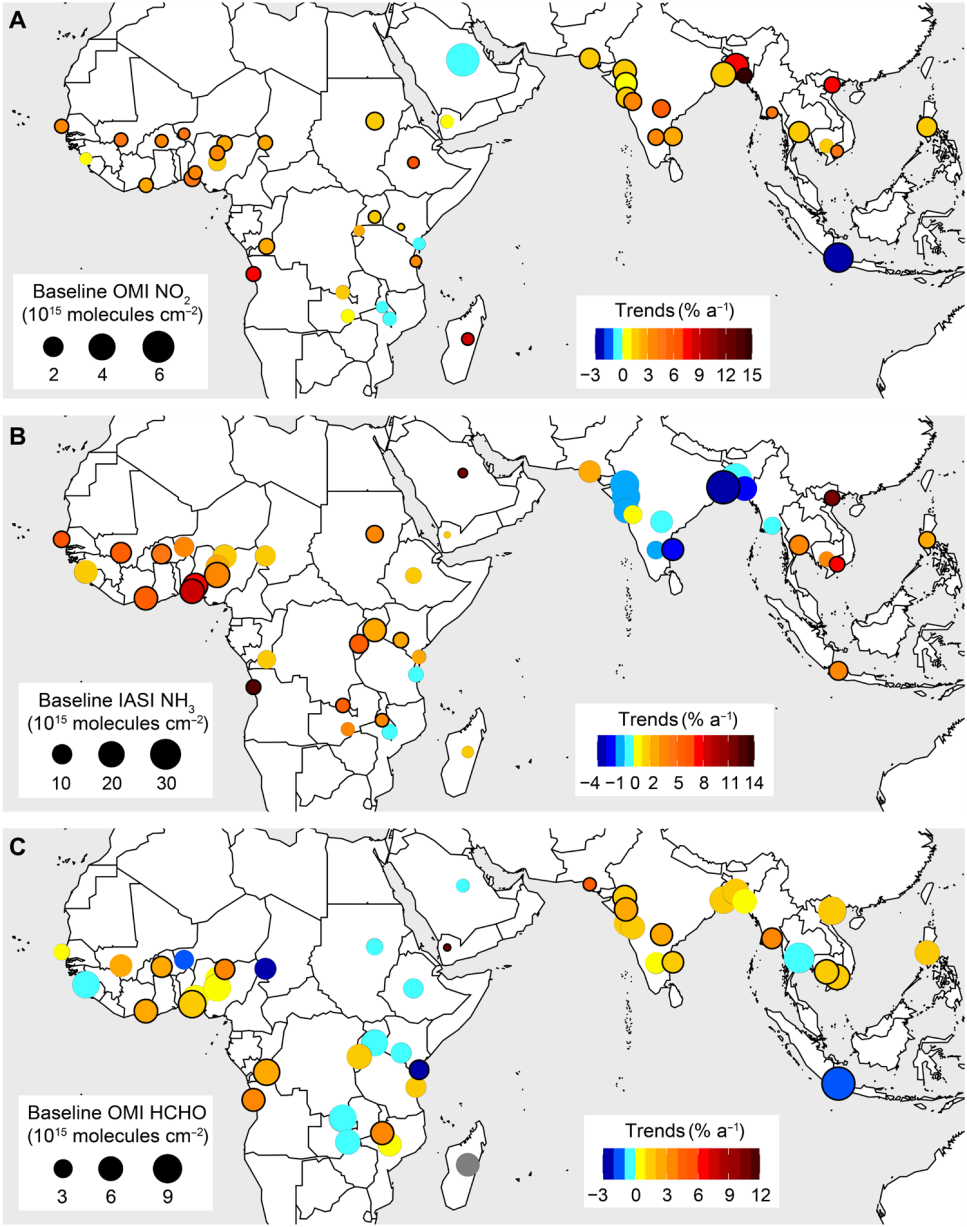
Trends in NO_2_, NH_3_, and reactive NMVOCs in rapidly growing cities in the tropics. (**A** to **C**) Trends in NO_2_ and reactive NMVOCs are for 2005–2018 and in NH_3_ for 2008–2018. Circle colors are the relative trends, and sizes are values at the start of the record (baseline). Outlines identify significant trends at the 95% confidence interval (CI). Warm colors indicate positive trends, and cool colors indicate negative trends. The trend in reactive NMVOCs at Antananarivo in Madagascar is gray because of the low temporal coverage. Trend values are in table S1. OMI, Ozone Monitoring Instrument; IASI, Infrared Atmospheric Sounding Interferometer.

Figure 2 shows trends in the short-lived pollutants NO_2_ for 2005–2018, NH_3_ for 2008–2018, and HCHO as a proxy for reactive nonmethane VOCs (NMVOCs) for 2005–2018 over these cities obtained from a long-term, consistent record of satellite observations (Table 1). Data processing to isolate the reactive component of the HCHO column is detailed in Materials and Methods. NO_2_ increases in almost all (41) cities, by 0.1 to 14.1% a^−1^. The increase is significant for 34 of these 41 cities (Fig. 2A) and has the potential to lead to increases in PM_2.5_ by forming aerosol nitrate. NO_2_ triples over Chittagong (Bangladesh) and more than doubles over Antananarivo (Madagascar), Hanoi (Vietnam), Luanda (Angola), and Dhaka (Bangladesh). NO_2_ declines in five cities, although the downward trend is only appreciable and significant for Jakarta (−2.0% a^−1^). This decline, already identified using an earlier record of observations from the same satellite instrument (*25*, *31*), likely reflects emission controls imposed on vehicles since 2005 (*25*, *32*). The record of NO_2_ for cities in Africa starts at ~60% lower values than Asian cities, due to prevalence of inefficient combustion sources with relatively low NO*_x_* emissions across Africa (*10*). In general, upward trends in NO_2_ cover a similar range in Africa (0.3 to 8.2% a^−1^) and Asia (0.8 to 7.7% a^−1^, excluding 14.1% a^−1^ for Chittagong). The directions of our NO_2_ trends are consistent with previous studies that have focused on large cities around the world (*25*, *27*, *33*–*35*). This consistency includes trend reversals from positive to negative over Jakarta and Riyadh in 2011 and from negative to positive over Manila in 2009 (*34*, *35*). The significant increases in NO_2_ in Northwest African cities, ranging from 2.0% a^−1^ over N’Djamena to 4.4% a^−1^ over Niamey and Lagos, are opposite to the large regional decline in NO_2_ of 4.5% a^−1^ reported by Hickman *et al.* (*16*) for the same instrument used here. These authors focused on the dry season (November to February) when intense and widespread open burning of biomass is prevalent.

**Table 1.** Satellite data products used to determine trends in NO_2_, reactive NMVOCs, NH_3_, and AOD for fast-growing cities in the tropics.

**Instrument**	**Launch** **platform**	**Launch** **date**	**Swath** **width**	**Measurement**	**Product** **name**	**Pixel** **resolution**	**Overpass** **time**	**Global** **coverage**	**Data** **retained**
OMI^*^	Aura	October 2004	2600 km	NO_2_	NASA SP v4.0^†^	13 km by 24 km^‡^	13 h30 LST^§^	Every 2 days^║,¶^	Cloud fraction < 50%
									Terrain reflectivity < 30%
									Solar zenith angle < 85°
				HCHO	QA4ECV v1.3^#^				Processing error flag = 0
									Processing quality lag = 0
									Cloud fraction < 50%
IASI^**^	Metop-A	October 2006	2130 km	NH_3_	v3R^††^	12 km^‡,‡‡^	09 h30 LST^§^	Daily^║^	Cloud fraction < 10%
MODIS^§§^	Aqua	May 2002	2230 km	AOD	Merged SDS C6.1^║║^	10 km by 10 km^‡^	13 h30 LST^§^	Near-daily^║^	Quality assurance flag = 3

*Ozone Monitoring Instrument.

†NASA Standard Product version 4.0 (https://doi.org/10.5067/Aura/OMI/DATA2017; last accessed 18 March 2021).

‡At nadir.

§Local solar time.

║Time to achieve global coverage for relevant satellite overpass time.

¶Global coverage degraded from daily in 2005–2009 to every 2 days thereafter due to the row anomaly.

#Quality Assurance for Essential Climate Variables version 1.3 (https://doi.org/10.18758/71021031; last accessed 18 March 2021).

**Infrared Atmospheric Sounding Interferometer.

††Reanalyzed IASI version 3.0.0 (https://iasi.aeris-data.fr/NH3R-ERA5_IASI_A_data/; last accessed 20 March 2021).

‡‡Circular pixels at nadir.

§§Moderate Resolution Imaging Spectroradiometer.

║║Merged Scientific Data Set Collection 6.1 (https://doi.org/10.5067/MODIS/MYD04_L2.061; last accessed 18 March 2021).

As with NO_2_, NH_3_ increases in almost all tropical cities in Africa, the Middle East, and Southeast Asia (Fig. 2B). Known dominant sources include agricultural activity, vehicles, and burning of waste and biomass in all regions, as well as large-scale industrial fertilizer production in Asia (*36*, *37*). The increase in NH_3_ over Jakarta while NO_2_ declines could reflect absence of air quality policies targeting agriculture and biomass burning (*26*). Decline in NH_3_ in almost all South Asian cities coincides with increases in precursor (SO_2_) emissions of acidic sulfate aerosols (*38*, *39*) that promote partitioning of NH_3_ to acidic aerosols to form PM_2.5_ (*40*). In India, SO_2_ emissions, mostly from industry and coal-fired power plants, are estimated to have increased by 50% from 2007 to 2016 (*41*). Aerosol partitioning of NH_3_ would also be promoted by an increase in abundance of acidic nitrate aerosol due to an increase in precursor emissions of NO*_x_*, supported by our positive trends in NO_2_ (Fig. 2A). Increase in abundance of chlorine from plastic waste fires would also promote aerosol uptake of NH_3_ (*42*), but there are no routine measurements to quantify trends in chlorine abundance. Increases in waste generation and absence of effective waste management support an increase in chlorine emissions from this source (*43*). Karachi is the lone city in South Asia with an increase in NH_3_ of 2.9% a^−1^. This may reflect increased use of synthetic fertilizers and excess NH_3_ from intense agricultural fires across the Indo-Gangetic Plain (*6*, *26*, *44*), although this increase is not significant. NH_3_ column densities at the start of the record are similar for Asia and Africa, due to a combination of similar sources (*36*, *37*, *44*). Our positive city trends are generally 1.5 to 2.5 times steeper than the trends at regional and national scales reported by Van Damme *et al.* (*26*). The steeper trends in cities could be due to greater abundance of urban sources of NH_3_ (*36*, *45*) and enhanced NH_3_ volatilization due to the urban heat island effect (*46*).

Trends in HCHO as a proxy for reactive NMVOCs (Fig. 2C) are not as homogeneous or significant as trends in NO_2_ and NH_3_, apart from Jakarta and cities in South Asia. Significant decline in reactive NMVOCs in Jakarta of 1.7% a^−1^ is similar to that for NO_2_ (Fig. 2A). This could be due to a decrease in vehicle exhaust emissions of unburned reactive hydrocarbons from enhanced efficiency of diesel vehicles due to greater use of biodiesel (*47*). Biodiesel usage in Indonesia has increased 16-fold from 2010 to 2019 (*48*). Our upward trends in reactive NMVOCs for Lagos (1.5% a^−1^), Mumbai (1.7% a^−1^), and Kinshasa (2.0% a^−1^) support sustained increases in reactive NMVOCs identified for 1997–2009 using an earlier record of satellite observations (*29*). The trend for Mumbai is not significant. The greatest increase in reactive NMVOCs occurs over Sana’a (>10% a^−1^), although the HCHO column densities there are close to the instrument detection limit and very small (<1 × 10^15^ molecules cm^−2^) at the beginning of the record after removing the background contribution (Materials and Methods). The population in Sana’a has increased by 4.7% a^−1^, but the country has also been embroiled in conflict and political instability, and the increase in the other pollutants is marginal (0.07 to 1.15% a^−1^) (table S1).

In nine African cities (Conakry, Niamey, N’Djamena, Addis Ababa, Nairobi, Kampala, Khartoum, Lubumbashi, and Lusaka), reactive NMVOCs decline at the same time as NO_2_ increases, consistent with a shift to more efficient combustion sources, as is suggested by regional anthropogenic emission inventories (*10*, *12*). Rates of increase in reactive NMVOCs in cities in South Asia of 0.6 to 4.3% a^−1^ are less steep than trends in NO_2_ (0.8 to 14.1% a^−1^). The generally positive, steeper, and greater number of significant trends in NO_2_ (and thus NO*_x_*) compared to those of reactive NMVOCs has implications for surface ozone formed from photochemical oxidation of NMVOCs in the presence of NO*_x_*. In most of the tropics, surface ozone is near the WHO 8-hour mean guideline of 100 μg m^−3^ (*49*) and, at these levels, is harmful to staple crops prevalent there (*50*). Ratios of total column HCHO (background + reactive NMVOCs) to tropospheric column NO_2_ (HCHO/NO_2_; fig. S4) in 2005 exceed values of 2 in almost all cities (reaching 23 in Conakry and Nairobi), indicative of chemical regimes in which ozone formation is sensitive to NO*_x_* sources. During the record of NO_2_ and HCHO observations used here (2005–2018), most of these cities are transitioning to a regime in which ozone production is sensitive to NMVOC sources, synonymous with many megacities in the United States, Europe, and China. If the future mimics the past, then the transition to sensitivity to NMVOCs could occur as soon as 2025 in Chittagong and Dhaka and 2030 in Addis Ababa, Hanoi, and Luanda. As NMVOCs are especially challenging to monitor and regulate (*6*, *51*), these trends suggest that there should be swift implementation of policies targeting NO*_x_* sources to halt the transition to a VOC-sensitive ozone production regime.

**Fig. 3.**
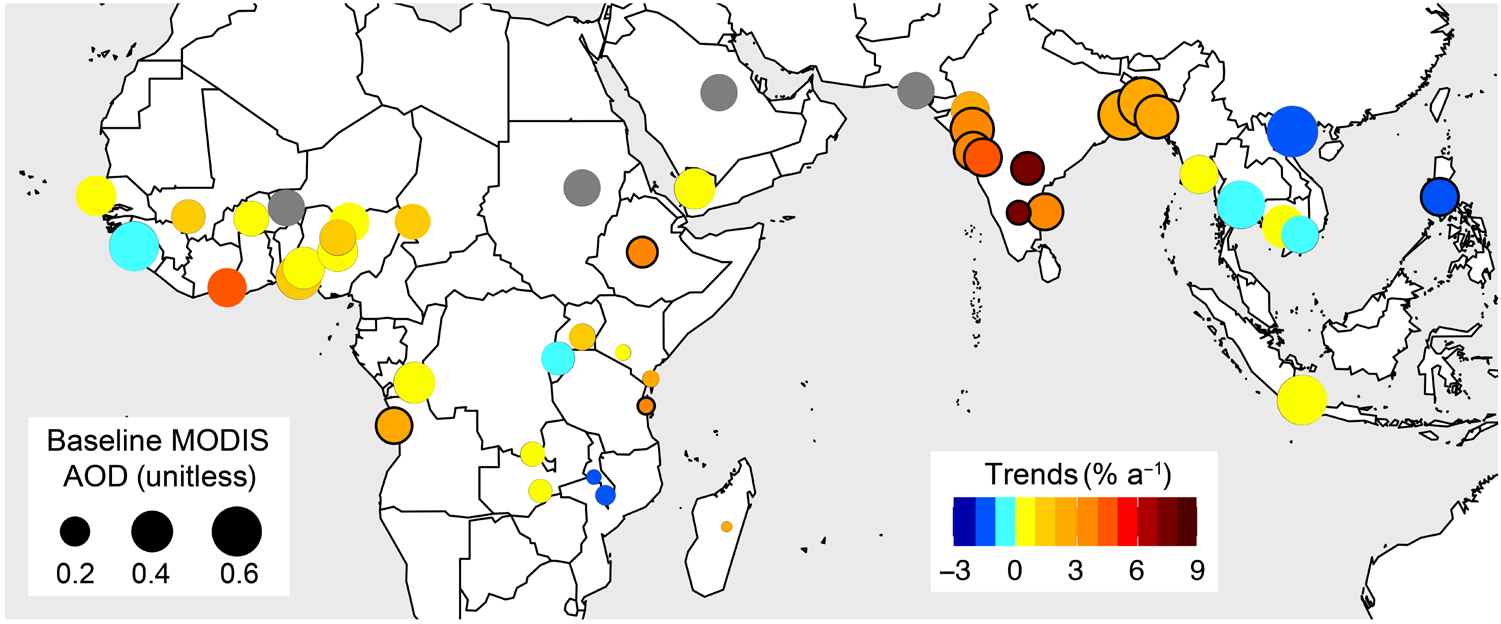
Trends in AOD as proxy for PM_2.5_ abundance in rapidly growing cities in the tropics for 2005–2018. Circle colors are relative trends in AOD, and sizes are values at the start of the record (baseline). Outlines identify significant trends at the 95% CI. Warm colors indicate positive trends, and cool colors indicate negative trends. Cities with poor temporal coverage are gray. AOD trend values are in table S1. MODIS, Moderate Resolution Imaging Spectroradiometer.

Figure 3 shows trends in AOD, which we interpret as trends in surface concentrations of PM_2.5_ (*6*). The relationship between AOD and PM_2.5_ is complicated by PM_2.5_ composition, vertical distribution of aerosols, relative humidity, cloud cover, and seasonality in planetary boundary layer dynamics and synoptic-scale meteorology (*52*–*55*). This causes inconsistencies in month-to-month variability in AOD and surface concentrations of PM_2.5_ but not in the long-term trends (*6*). AOD at the start of the record is >0.25 over cities in West Africa, the Middle East, South Asia, and Southeast Asia due to a mix of large aerosol sources such as biomass burning and vehicular emissions in all these regions; desert dust in West Africa, the Middle East, and South Asia; and large industrial sources in Asia (*56*–*58*).

**Fig. 4.**
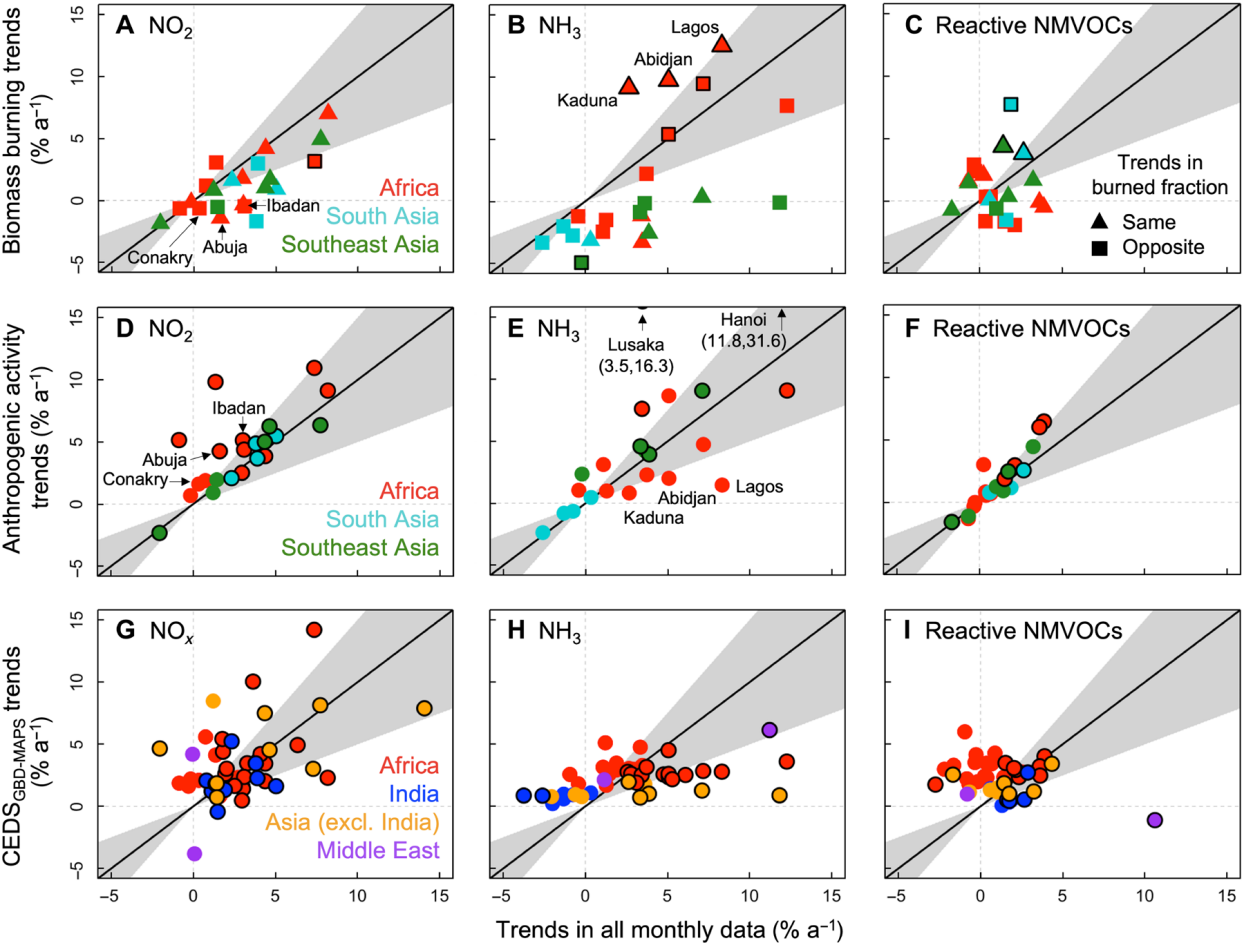
Constraints on factors influencing trends in short-lived air pollutants in rapidly growing cities in the tropics. Panels compare trends in all data (Fig. 2) to trends in biomass burning (**A** to **C**) and anthropogenic activity (**D** to **F**) for 22 cities influenced by biomass burning (fig. S3 and Materials and Methods) and to trends in bottom-up anthropogenic emissions (**G** to **I**) for all 46 cities. Shape colors distinguish regions, and, in (A) to (C), shape types indicate trends that are consistent with (triangles) or opposite to (squares) the trends in burned fraction (fig. S3). Outlines identify significant trends at the 95% CI for biomass burning (A to C), anthropogenic activity (D to F), and all data (G to I). Trends are significant for Lusaka and Hanoi in (E), which are off scale. The gray shading is the ±50% spread around the black 1:1 line. Dotted lines discern positive and negative trends.

Trends in AOD from 2005 to 2018 in South Asian cities are steep (2.5 to 7.8% a^−1^) and significant. AOD more than doubles in Bangalore (7.8% a^−1^) and Hyderabad (7.3% a^−1^). Earlier studies have reported similar positive trends for these cities (*28*, *39*), so our contemporary record supports sustained rapid growth in AOD (and thus PM_2.5_). Desert dust likely does not contribute to trends in AOD over South Asian cities, as desert dust optical depth has declined over the Thar Desert and makes a negligible contribution to AOD trends across the rest of India (*59*). This suggests that the substantial increase in PM_2.5_ in South Asia is due to increased formation of secondary inorganic aerosols resulting from increases in SO_2_ emissions forming aerosol sulfate (*38*, *39*), NO*_x_* emissions forming aerosol nitrate (Figs. 2A and 4G), and buffering by ammonium formed when NH_3_ partitions to these acidic aerosols, which leads to declining NH_3_ abundance (Fig. 2B). The increase in reactive NMVOCs (Fig. 2C), which includes precursors of secondary organic aerosols, likely also contributes to the increase in PM_2.5_.

Trends in cities in other regions are not as consistent as for South Asia. In Southeast Asia, only Manila shows a significant decline in PM_2.5_ (−2.0% a^−1^), while, in Africa, there are significant increases in Luanda (2.3% a^−1^), Addis Ababa (3.3% a^−1^), and Dar es Salaam (3.3% a^−1^). The significant increase in AOD for Addis Ababa may be influenced by the positive trend in desert dust optical depth over Ethiopia (*59*). PM_2.5_ increases in Jakarta despite a significant decrease in precursor emissions of NO*_x_* (Fig. 2A) and reactive NMVOCs (Fig. 2C). This may be due to an increase in sulfate in Jakarta from high sulfur fuel combustion and regional adoption of coal-fired power generation (*60*, *61*) as well as lack of policies targeting biomass burning and agricultural sources. Our positive trends in AOD for most West African cities are opposite to the reported weak regional decline in AOD of 0.1% a^−1^ (*62*).

### Disentangling underlying sources influencing trends in air quality

To assess whether conventional biomass burning or emerging anthropogenic sources dominate tropical city trends in air quality, we separate the satellite data into biomass burning and nonbiomass burning data, compare trends in these categories to trends in all the data (Fig. 2), and confirm that the nonbiomass burning data are dominated by anthropogenic activity for pollutants that also have large natural sources. The trend comparison is shown in Fig. 4 for the 22 tropical cities that we identify to be substantially influenced by biomass burning (Materials and Methods). In general, there are fewer significant trends in biomass burning (Fig. 4, A to C) than there are in anthropogenic activity (Fig. 4, D to F). The Fig. 2 trends in the reactive NMVOC portion of the HCHO column appear to be predominantly influenced by anthropogenic activity. This is supported by either the same or steeper trends in anthropogenic activity data than all data (Fig. 4F), by lack of consistent trends for all but one city in the biomass burning comparison (Fig. 4C), and by absence of significant trends in biogenic VOCs determined by sampling HCHO in surrounding rural areas that should be dominated by biogenic VOCs (in particular isoprene) (Supplementary Text).

Anthropogenic activity appears to be the driving force for trends in NO_2_ in most cities (Fig. 4D), although some cities have similar positive trends in biomass burning and all data (shapes along the 1:1 line in Fig. 4A). Six African cities exhibit very steep increases in anthropogenic activity NO_2_ of 1.6 to 9.8% a^−1^ (points above the gray shading in Fig. 4D). Three of these, all in West Africa (Abuja, Ibadan, and Conakry), lie below the gray shading in Fig. 4A, suggesting that negative or muted trends in biomass burning dampen the effect of increasing anthropogenic activity on degradation in air quality. This is consistent with findings from the regional study by Hickman *et al.* (*16*) and supported by a decline in burned fraction for the two cities in Nigeria (Abuja and Ibadan; fig. S3). Regional bottom-up inventories also suggest that anthropogenic emissions already or will soon rival those from biomass burning (*10*, *12*).

The NH_3_ trends (Fig. 4, B and E) exhibit a compensating effect between anthropogenic activity and biomass burning in many cities. Six cities with steep increases in anthropogenic activity of 1.0 to 31.6% a^−1^ have negative trends in biomass burning, and three West African cities (Lagos, Kaduna, and Abidjan) with steep increases in biomass burning have much shallower trends in anthropogenic activity. These same three cities exhibit positive trends in burned fraction but not in biomass burning NO_2_ (Fig. 4A) or reactive NMVOCs (Fig. 4C). This may be due to a shift in the type of vegetation burned (*63*), a transition to more anoxic fires favoring NH_3_ over NO*_x_* and longer-lived hydrocarbons over reactive oxygenated VOCs, or due to greater prevalence in the dry season of anthropogenic NH_3_ sources such as waste burning. Evidence of these shifts requires further investigation.

We also assess the skill of a contemporary anthropogenic emission inventory at reproducing trends in Fig. 2 for all 46 cities (Fig. 4, G to I), as satellite observations provide constraints on precursor emissions of short-lived pollutants (*25*, *30*, *37*) and because we find that anthropogenic activity dominates trends in air quality in tropical cities. We use the global contemporary CEDS_GBD-MAPS_ inventory (Materials and Methods) that has been applied in the Global Burden of Disease (GBD) studies (*64*). CEDS_GBD-MAPS_ NO*_x_* emission trends reproduce the direction of trends in satellite NO_2_ for most cities (Fig. 4G), although, for many, the discrepancy exceeds 50%. CEDS_GBD-MAPS_ does not capture the policy-driven decline in emissions in Jakarta (+4.7% a^−1^ in CEDS_GBD-MAPS_ and −2.0% a^−1^ in the observations) or the marginal, although nonsignificant, increase in NO*_x_* in Sana’a (−3.8% a^−1^ in CEDS_GBD-MAPS_ and +0.07% a^−1^ in the observations). According to CEDS_GBD-MAPS_, the steepest increases in NO*_x_* emissions in African cities are from nonresidential energy generation (4.6% a^−1^) and industry (4.2% a^−1^). These sources together account for 65 to 70% of CEDS_GBD-MAPS_ NO*_x_* in Indian cities, but the steepest increases are for off-road transport (8.6% a^−1^) and a mix of nontraditional combustion sources (2.8% a^−1^). In Asian cities, excluding India, the largest growth in CEDS_GBD-MAPS_ NO*_x_* is from the dominant sectors’ energy generation (4.2% a^−1^), industry (5.3% a^−1^), and off-road transport (6.3% a^−1^).

Changes in NO*_x_* lifetime and the relative contribution of the free tropospheric portion of the column may also contribute to divergence in column and emission trends. Most studies assessing these effects have focused on cities in the northern hemisphere that have experienced substantial policy-driven decline in NO*_x_* emissions (*65*, *66*). Results from these studies suggest that NO*_x_* lifetime and background changes as NO*_x_* emissions increase in VOC-rich environments typical of the tropics (fig. S4) should have opposing effects on agreement between trends in NO_2_ columns and NO*_x_* emissions. Trends in the column are likely steeper than trends in emissions because of decline in NO*_x_* lifetime, whereas trends in the NO_2_ column should be dampened relative to trends in emissions because of decline in the relative contribution of the NO*_x_* background.

Trends in CEDS_GBD-MAPS_ NH_3_ emissions are positive for all cities, but two to five times less than the observed trends (Fig. 4H). Underestimates in CEDS_GBD-MAPS_ trends are greatest for all but two of the cities that we identified to be strongly influenced by steep increases in anthropogenic sources (Fig. 4E). Similarly large underestimates in emission inventory trends have been reported at the regional and national scale (*26*). Inventories may underestimate rapid changes in agricultural activity, such as increases in livestock numbers and adoption of synthetic nitrogen fertilizers (*67*, *68*). Growth in agricultural productivity in sub-Saharan Africa is almost double the global average (*68*). Waste burning is another prevalent source of NH_3_. Waste generation is projected to increase by 2 to 6% a^−1^ across the tropics over the next three decades (*69*). Much of this will be burned if ineffective waste management persists. Divergence of NH_3_ trends in Indian cities and nearby Asian cities is due to the increase in acidic aerosols already discussed. According to CEDS_GBD-MAPS_, NH_3_ emissions in Indian cities grew at a rate of 1% a^−1^, mostly due to a 2% a^−1^ increase in agricultural emissions. Discrepancies in observed and CEDS_GBD-MAPS_ NH_3_ in Riyadh and Sana’a are large, but emissions for these cities are small [3 to 10 kilotons (kt) a^−1^ during 2005–2017] compared to the other tropical cities (30 to 80 kt a^−1^ for the same time period) and mainly come from waste processing.

Trends in bottom-up and top-down reactive NMVOCs are generally similar for cities with significant increases in these and are dominated by increases in residential energy combustion in CEDS_GBD-MAPS_. Trends diverge for Sana’a (−1.1% a^−1^ for CEDS_GBD-MAPS_ and +10.6% a^−1^ for the observations), but observed values are small and may be prone to error. As with NO_2_, CEDS_GBD-MAPS_ trends in reactive NMVOCs in Jakarta (+2.6% a^−1^) are opposite to the observations (−1.7% a^−1^). Significant decline in reactive NMVOCs over Mombasa in the observations (−2.7% a^−1^), but not in the inventory (+1.8% a^−1^), may be due to a shift toward combustion-efficient sources due to substantial development of the Mombasa seaport to meet increasing demand for imported goods (*70*), although this is not supported by the nonsignificant decline in NO_2_ (0.3% a^−1^; Fig. 2A).

### Increase in premature deaths from enhanced exposure to air pollutants hazardous to health

Urban population in the tropical cities in Fig. 1 increased on average by 2 to 10% a^−1^ in Africa, 4 to 5% a^−1^ in the Middle East, 1 to 7% a^−1^ in South Asia, and 1 to 8% a^−1^ in Southeast Asia from 2005 to 2018. Rapid population growth combined with steep and significant increases in NO_2_ in most cities (Fig. 2A) and in PM_2.5_ in all cities in South Asia and many in Africa (Fig. 3) will substantially increase population exposure to hazardous pollutants. The effects of long-term exposure to ambient air pollution includes well-established health outcomes such as respiratory and cardiovascular diseases (*71*, *72*) and recently identified health end points such as dementia, impaired cognition, and loss of fertility and eyesight (*73*–*77*). Premature mortality due to exposure to ambient PM_2.5_ from anthropogenic sources is already substantial in Asia but is relatively low in Africa, particularly in comparison to communicable diseases and exposure to indoor air pollution and ambient pollution from natural sources (*19*, *20*, *64*, *71*). Hickman *et al.* (*16*) suggest, using the same NO_2_ observations, that regional air quality in Africa has improved because of decline in biomass burning activity. Their analysis, however, focused on the burning season and did not account for rapid growth in urban population, enhancing population exposure to air pollution.

**Fig. 5.**
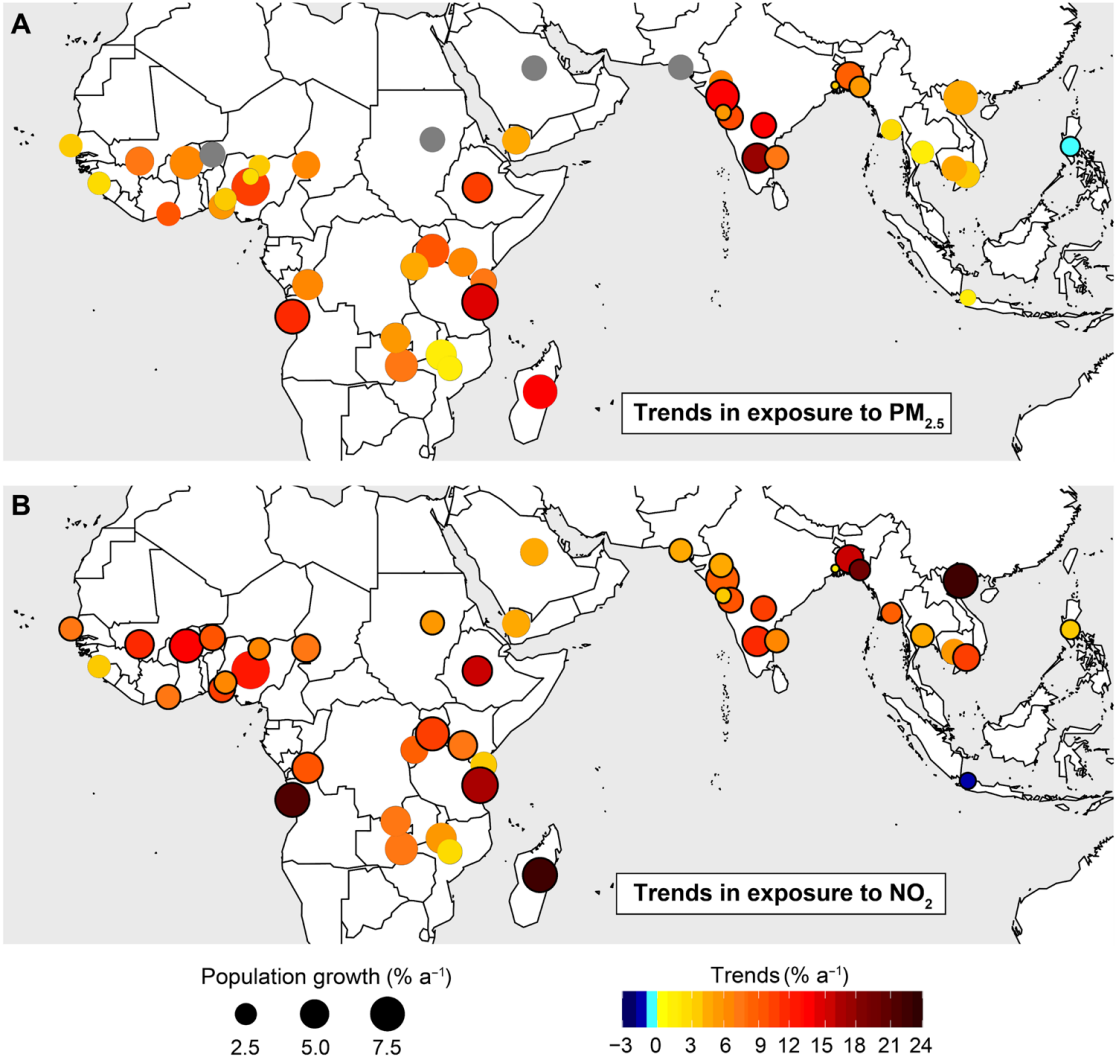
Trends in exposure to air pollutants hazardous to health in rapidly growing tropical cities. Circle colors are trends in exposure to PM_2.5_ (**A**) and NO_2_ (**B**). Circle sizes are annual average increases in urban population from 2005 to 2018. Outlines identify cities with significant trends in PM_2.5_ (Fig. 3) and NO_2_ (Fig. 2A) at the 95% CI. Cities with low temporal coverage in AOD are gray.

The trends in exposure to PM_2.5_ and NO_2_ that we obtain for the 46 tropical cities (Materials and Methods) are shown in Fig. 5. Population exposure to PM_2.5_ increases in all cities except Manila (−0.8% a^−1^), where a 2.0% a^−1^ decline in PM_2.5_ (Fig. 3) counters an equivalent increase in population. Exposure to NO_2_ increases in all cities except Jakarta (−1.2% a^−1^), although the absolute health gains due to decline in exposure to NO_2_ in Jakarta will be more than offset by a 1.8% a^−1^ increase in exposure to PM_2.5_ due to a 1.3% a^−1^ increase in population and a small, although not significant, increase in PM_2.5_ (0.5% a^−1^; Fig. 3). In general, upward trends in exposure are 1.5 to 3.0 times steeper than the positive trends in PM_2.5_ (Fig. 3) and NO_2_ (Fig. 2A) alone. Increases in population exposure to PM_2.5_ are 3.1 to 17.9% a^−1^ in South Asian cities and >10% a^−1^ in Addis Ababa (10.6% a^−1^), Abuja (10.7% a^−1^), Antananarivo (13.0% a^−1^), Luanda (11.7% a^−1^), and Dar es Salaam (14.8% a^−1^) in Africa. These same African cities also experience the steepest increases in NO_2_ exposure of >12% a^−1^. Across Africa, the increase in exposure to NO_2_ ranges from 2.0% a^−1^ in Blantyre to 21.3% a^−1^ in Luanda and 23.0% a^−1^ in Antananarivo.

**Fig. 6.**
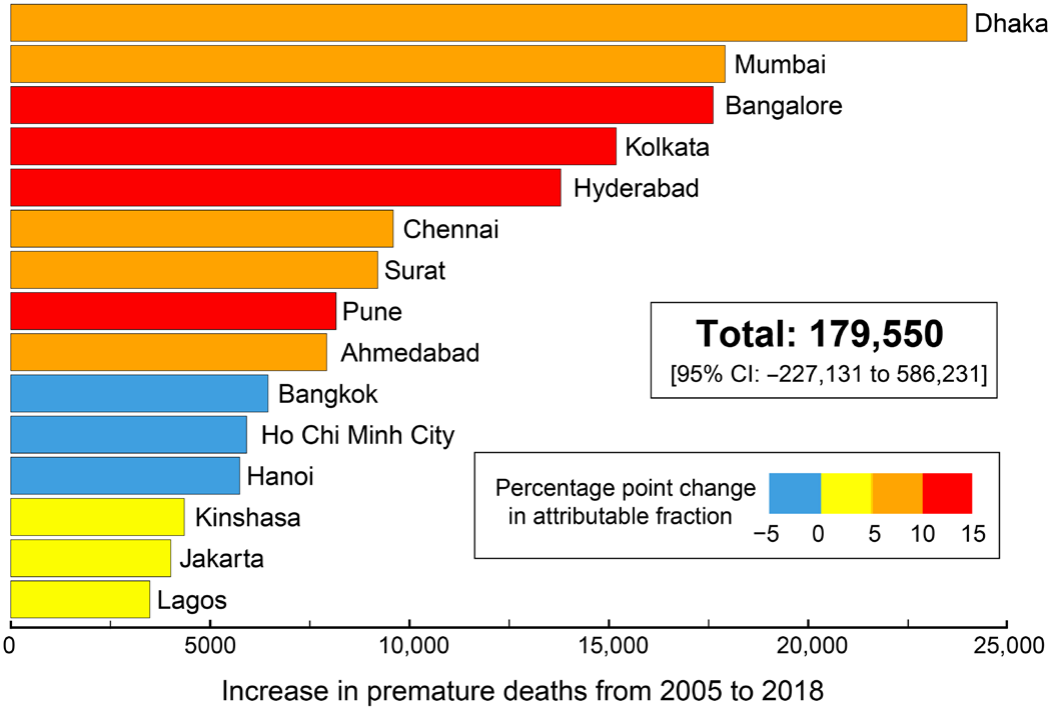
Increase in premature deaths due to increase in exposure to PM_2.5_ in rapidly growing tropical cities. Bars give the increase in premature mortality in 2018 relative to 2005 for the top 15 tropical cities, colored by the percentage point change in fraction of premature deaths attributable to exposure to PM_2.5_. Inset value is the total for the cities in Fig. 1 with detectable trends in AOD (Fig. 3). Values for all cities are in table S2, and the relative contribution of individual factors (PM_2.5_, population, and baseline mortality) is in fig. S5.

Figure 6 ranks the 15 cities with the largest increases in the number of premature deaths over the 14-year observing period, mostly in Asia (13 of 15). Premature deaths range from 3500 in Lagos to 24,000 in Dhaka. Values for each city are in table S2. These are obtained by calculating absolute PM_2.5_ in each city using our annual trends in AOD (Fig. 3) and PM_2.5_ from a chemical transport model for a single year at the center of the observing period (2012) and applying these to a health risk assessment model that relates the risk of premature mortality to ambient PM_2.5_ (Materials and Methods). We focus on PM_2.5_ because of its much greater health risk than NO_2_ (*78*, *79*). Increased incidence of morbidity will also result from the substantial increases in population exposure to PM_2.5_ and NO_2_ (Fig. 5). The increase in premature mortality, summed over the cities in Fig. 1 with discernible trends in AOD (Fig. 3), is 180,000 [95% confidence interval (CI): −230,000 to 590,000], an average increase of 13,000 each year. This is a 62% increase in premature deaths due to PM_2.5_ exposure, from 290,000 (95% CI: 200,000 to 370,000) in 2005 to 470,000 (95% CI: 70,000 to 870,000) in 2018. The contribution to total premature deaths increases from 27% in 2005 to 32% in 2018, and the number of cities where at least one-third of deaths is attributable to exposure to PM_2.5_ increases from 14 in 2005 to 20 in 2018. PM_2.5_ exposure persists as the leading cause (>50%) of premature deaths in Kano (Nigeria), N’Djamena (Chad), and Kolkata (India).

Premature mortality estimates for 2005 and for 2008 are significant for almost all cities (table S2). The wide range in the premature mortality 95% CIs is due to large uncertainty in the health risk assessment model for annual mean PM_2.5_ > 50 μg m^−3^ (*19*, *79*), typical of most cities in South Asia and West Africa. As the 95% CIs for 2005 and 2018 overlap, changes in premature mortality are not significant for most cities (as indicated in table S2) and in the total given in Fig. 6. Kolkata makes the largest contribution to uncertainties in the total, as PM_2.5_ there is much greater than 50 μg m^−3^, increasing from 78.8 μg m^−3^ in 2005 to 106.3 μg m^−3^ in 2018 (table S2). The total increase in premature deaths from 2005 to 2018 without Kolkata is 165,000, and the lower limit of the CI exceeds zero (95% CI: 40,000 to 290,000).

We also determine the contribution of individual factors (PM_2.5_, population, and baseline mortality) to changes in total premature mortality (Materials and Methods, fig. S5, and table S3). African cities experience substantial decline in baseline mortality that reduces the impact of increases in population and PM_2.5_ on premature mortality. Decline in baseline mortality more than offsets increases in the other factors in Blantyre, Lilongwe, and Kaduna, leading to decline in premature mortality attributable to PM_2.5_. The significant 3.1% a^−1^ increase in NO_2_ in Kaduna may still lead to severe public health outcomes. The increase in premature mortality in South Asian cities is due exclusively to population and PM_2.5_. Although trends in premature mortality are positive for all Southeast Asian cities, the relative contribution of each factor is mixed. The contribution of factors in Phnom Penh and Yangon is similar to African cities. Decline in PM_2.5_ in Bangkok, Hanoi, Ho Chi Minh City, and Manila does not mitigate the influence of increases in population and baseline mortality.

Our premature mortality estimates for 2005 and 2018 are on median a factor of 3 more than those reported for the same cities in studies that determine premature mortality using the Integrated Exposure-Response (IER) model that is also applied to GBD studies (*80*–*83*). The IER model uses health data from active smoking, secondhand tobacco smoke, and indoor air pollution to derive a relationship between outdoor PM_2.5_ exposure and risk of premature mortality. Updated health risk assessment models that no longer rely on proxies for ambient PM_2.5_ and incorporate data from global cohort studies, such as the Global Exposure Mortality Model (GEMM), yield premature mortality estimates that are more than double those from the GBD (*71*). We use an updated health risk assessment model (*79*) that is derived with more cohort studies and health end points covering a wider age range (14 years and older) and PM_2.5_ concentration range than both the IER model and the GEMM (*71*, *83*). In our previous work, we showed that this new health risk assessment model yields premature mortality estimates that are 50% more than those estimated with the GEMM (*19*). The GEOS-Chem model version that we use typically overestimates surface PM_2.5_ by 5 to 10% estimated from comparison to surface observations for North America and Europe and satellite-derived PM_2.5_ for Africa (*19*, *84*). If we artificially decrease modeled PM_2.5_ by 10%, then we find that the additional premature mortality from 2005 to 2018 only changes by 6%. Decreasing from 180,000 deaths to 170,000.

## DISCUSSION

With our targeted sampling of satellite observations over 46 densely populated fast-growing tropical cities, we find large and significant increases in NO_2_, NH_3_, reactive VOCs, and AOD (and thus PM_2.5_) in these cities at rates two to three times faster than or opposite in sign to national and regional trends. The degradation in air quality in the past two decades is mostly influenced by anthropogenic activity rather than traditional widespread open burning of biomass. Only Jakarta shows evidence of air quality improvements due to policy measures, and those improvements have had a limited effect, leading to decline in NO_2_ and reactive VOCs but not in NH_3_ or PM_2.5_.

We also determine that ozone formation is on track to transition from strongly NO*_x_*-sensitive to the more challenging to regulate VOC-sensitive regime. In some cities, this may occur as early as 2025. We find large increases in population exposure to hazardous air pollutants PM_2.5_ and NO_2_ and estimate 13,000 additional premature deaths each year from this increase in population exposure to air pollution. These trends will almost certainly be sustained in the future because of projected population growth (Fig. 1) and absent effective air quality policies.

The predicted population for 2100 predates the coronavirus disease 2019 (COVID-19) pandemic, but unemployment, inequitable health care access, and reduced fertility resulting from the pandemic (*85*, *86*) are only likely to delay rather than prevent the deleterious effects of exposure to air pollution that our findings suggest. The pandemic has demonstrated that health care systems in tropical countries are vulnerable to the looming health crisis supported by our exposure trends. Immediate and strict policy measures are needed to improve air quality and curtail increased exposure to hazardous pollutants due to abrupt population growth and urbanization in a part of the world that accounts for an increasingly large portion of the global population.

## MATERIALS AND METHODS

### Satellite datasets, city sampling, and trend estimates

We use Earth observations of tropospheric column NO_2_ and total column HCHO from the Ozone Monitoring Instrument (OMI), attenuation of light by aerosols throughout the atmospheric column or AOD from the Moderate Resolution Imaging Spectroradiometer (MODIS), and total column NH_3_ from the Infrared Atmospheric Sounding Interferometer (IASI) to determine trends in air quality, precursor emissions, and population exposures over the rapidly growing tropical cities identified in Fig. 1. The record of observations we use is for 2005–2018 from OMI and MODIS and for 2008–2018 from IASI. Table 1 provides additional details of the instrument features, satellite data products, and data quality flags used to process the data. In our previous work, we demonstrated that these space-based observations of tropospheric column NO_2_ and total column NH_3_ reproduce month-to-month variability in surface concentrations of these and that satellite observations of AOD reproduce long-term trends in surface observations of PM_2.5_ (*6*). The same assessment of the skill of satellite observations of HCHO in reproducing changes in surface concentrations of reactive NMVOCs was not possible, as measurements of reactive NMVOCs are sparse in space and time and routine measurements are limited to hydrocarbons.

We calculate city-wide monthly means by sampling satellite pixel centers that fall within the city boundaries using shapefiles mostly from the Database of Global Administrative Areas (GADM) version 3.6 (https://gadm.org/; last accessed 12 March 2021). Satellite data coverage can be low for smaller cities, exacerbated by persistent clouds in the tropics. We address this by extending the sampling domain beyond the city boundaries relative to the instrument pixel size (6.5 km for OMI and MODIS and 10 km for IASI), as in (*6*), for the 22 smallest tropical cities (indicated in table S1). In our analysis, we only retain months with at least 5 pixels, as in (*6*).

We isolate the contribution of local HCHO sources (direct emissions and oxidation of reactive NMVOCs) to total column HCHO by subtracting the background column component due to oxidation of methane and other long-lived VOCs (*6*, *30*). We do this by calculating monthly mean background columns over remote ocean domains closest to the cities of interest that extend over the same latitudinal range as the selected cities (fig. S2), where feasible. This ensures consistent seasonality between the background column and columns over the target cities. We then apply the nonlinear fit described in (*87*) that accounts for seasonality in the time series to the monthly mean background HCHO and subtract the fitted values from the city-wide monthly means, as in (*30*) and (*6*). We also assess the contribution of biogenic VOCs to trends in reactive NMVOCs by sampling HCHO over a 0.2° by 0.2° rural area 50 to 100 km away from the city.

All atmospheric components of interest in this work exhibit a distinct seasonality in the tropics due to seasonality in photochemistry, planetary boundary layer dynamics, synoptic meteorological events such as monsoons, and sources such as biomass burning (*6*, *26*, *88*). To account for this in the trend analysis, we fit the nonlinear function from van der A *et al.* (*87*) to the time series of city-wide monthly means. This is only applied to cities with >30% temporal coverage (>50 months for OMI and MODIS and >40 months for IASI). Cities with too few monthly means are shown as gray circles in Figs. 2 and 3, and fig. S4. Trends are considered significant at the 95% CI (*P* < 0.05) if the CI range does not intersect zero.

### Trends in biomass burning and anthropogenic activity

Intense regional open burning of biomass contributes to large seasonal enhancements in air pollution in the tropics, and anthropogenic activity dominates the nonbiomass burning period (*7*, *9*, *89*). To determine whether biomass burning or anthropogenic activity influences trends in NO_2_, NH_3_, and reactive NMVOCs (Fig. 2), we separate city-wide monthly means into months above and below the 75th percentile in each year. We find that months above 75th percentile values coincide with months known to be influenced by biomass burning in 22 of the cities in Fig. 1 (indicated in fig. S3). These are December to March in Northern Africa; July to November in Southern Africa (*90*); January to April in South Asia and Southeast Asia, north of the Equator; and August to October in Southeast Asia, south of the Equator (*17*, *91*). We remove from these biomass burning months the contribution of anthropogenic sources determined as the annual means of the monthly data that fall below the 75th percentile for the corresponding year. We refer to the resultant values as biomass burning data and the values below the 75th percentiles as nonbiomass burning or anthropogenic activity data. We also assess consistency in trend directions between biomass burning data obtained with our statistical approach and biomass burning activity as indicated by satellite-derived burned fraction. The burned fraction product that we use is the Global Fire Emissions Database (GFED) version 4.1 that includes improved detection of small fires (v4.1s; https://globalfiredata.org/pages/data/; last accessed 22 April 2021) (*92*). This is provided at 0.25° by 0.25° for 2005–2016. We calculate annual burned fraction over the 22 target cities by sampling GFEDv4.1s grid cells that overlap with the same sampling extent as the satellite observations and calculate trends in the biomass burning data, nonbiomass burning (anthropogenic activity) data, and annual burned fraction using the linear Theil-Sen median estimator (*93*, *94*). We use the robust Theil-Sen median estimator as it reduces the influence of temporal variability in the data on the trend estimates.

### Trends in bottom-up estimates of anthropogenic emissions

Satellite observations of the relatively short-lived pollutants NO_2_ (lifetime of ~6 hours against conversion to reservoir compounds), HCHO (lifetime of 2 to 3 hours), and NH_3_ (lifetime of 2 to 15 hours) provide constraints on precursor emissions of NO*_x_*, reactive NMVOCs, and NH_3_ (*25*, *30*, *37*, *88*). We assess the representation of these in bottom-up estimates of anthropogenic emissions and characterize possible anthropogenic sources contributing to the observed trends using the CEDS_GBD-MAPS_ inventory (https://doi.org/10.5281/zenodo.3754964; last accessed 20 March 2021) developed for the U.S. Health Effects Institute (HEI) Global Burden of Disease–Major Air Pollution Sources (GBD-MAPS) project. It extends the record of emissions of the original Community Emissions Data System (CEDS) inventory (*95*) from 1970–2014 to 1970–2017 and improves representation of regional emissions by updating activity data and emission factors with data from other global, regional, and national inventories (*96*). Anthropogenic emissions in CEDS_GBD-MAPS_ are provided as monthly gridded (0.5° by 0.5°) values for 11 broad source sectors (*96*). We sum 16 of 23 NMVOC classes with atmospheric lifetimes of <2 days to represent reactive NMVOC emissions. We isolate annual total city emissions from CEDS_GBD-MAPS_ by sampling grid cells in the same way as we do GFEDv4.1s burned fraction and calculate trends using the same Theil-Sen median estimator (*93*, *94*). Emissions of NO*_x_* and reactive NMVOCs are compared to trends in OMI NO_2_ and the reactive NMVOC component of OMI HCHO, respectively, for 2005–2017. Emissions of NH_3_ are compared to trends from IASI NH_3_ for 2008–2017.

### Trends in population exposure to hazardous air pollutants

We determine trends in city population exposure to PM_2.5_ and NO_2_ by calculating annual city population pseudo-exposure to total column AOD and tropospheric column NO_2_ using the standard population exposure (*E*_pop_) formula

$$E_{\text{pop}}=\sum_{i=1}^{N}C$$ (1)

where *N* is the total population from the United Nations (UN) (*1*) for each tropical city in Fig. 1 and *C* is the annual mean of the nonlinear fit used in that city to estimate trends in total column AOD as proxy for surface PM_2.5_ (Fig. 3) and in tropospheric column NO_2_ as proxy for surface NO_2_ (Fig. 2A). We calculate *E*_pop_ at the record start (2005) and end (2018) to estimate relative trends in exposure. This approach draws on our previous findings that trends in satellite observations of column values are consistent with trends in surface concentrations (AOD for surface PM_2.5_ and tropospheric column NO_2_ for surface NO_2_), even for cities where seasonality in AOD and PM_2.5_ are decoupled (*6*).

### Premature mortality from exposure to ambient PM_2.5_

We estimate the increase in premature mortality due to the increase in exposure to PM_2.5_ using annual mean PM_2.5_ calculated with the GEOS-Chem model at the midpoint (2012) of the time period that we use here (2005–2018) and a risk assessment model that relates PM_2.5_ exposure to risk of premature mortality. A description and validation of GEOS-Chem PM_2.5_ is in (*19*). Briefly, GEOS-Chem simulations were carried out at fine spatial scales of 0.5° by 0.67° (~50 km by 67 km) over Asia and Africa using a comprehensive suite of natural and anthropogenic emissions, dynamic meteorology, and detailed coupled gas and aerosol phase chemistry (*19*). We calculate mean PM_2.5_ for 2012 in each city by sampling GEOS-Chem grid cells in the same way as we do GFEDv4.1s burned fraction and CEDS_GBD-MAPS_. We then use these values with the trends in AOD (Fig. 3) as proxies for trends in surface PM_2.5_ to determine concentrations of PM_2.5_ in 2005 and 2018. The health risk assessment model that we use is from a recent meta-analysis (*79*) of cohort studies for people 15 years and older and covering more extensive geographies, a wider PM_2.5_ concentration range, and more health end points than previous approaches (*19*, *79*). We use the health risk assessment model to calculate the fraction of all-cause premature deaths attributable to exposure to PM_2.5_ in 2005 and 2018 using the GEOS-Chem and satellite AOD–derived PM_2.5_ as input. We then convert this to the number of premature deaths attributable to exposure to PM_2.5_ for people older than 14 years in each city in 2005 and 2018 using country-level age-specific baseline mortality rates from the GBD (*97*), population data for each city from the UN (*1*), and data on the proportion of the population in each country >14 years old from the World Bank (*98*). The difference in the resultant premature deaths then gives the change in premature mortality from 2005 to 2018 due to the combined effect of changes in exposure to PM_2.5_, to population, and to baseline mortality rates. We also quantify the relative role of each of these factors as the log transform of the ratio of premature mortality obtained with each of these parameters held fixed at 2005 values to premature mortality due to the combination of all factors, as in (*81*).

## Supplementary Materials

### This PDF file includes:

Supplementary Text Figs. S1 to S5 Tables S1 to S3 References
