# Supplementary material for: Rapid rise in premature mortality due to anthropogenic air pollution in fast-growing tropical cities from 2005 to 2018
Source: Sci Adv. 2022 Apr 8;8(14):eabm4435. doi: 10.1126/sciadv.abm4435 (PMC8993110; doi:10.1126/sciadv.abm4435)
Supplement: Supplementary file 1 — Supplementary Text Figs. S1 to S5 Tables S1 to S3 References [file sciadv.abm4435_sm.pdf]

Science Advances
AAAS

Supplementary Materials for

Rapid rise in premature mortality due to anthropogenic air pollution in fast-growing tropical cities from 2005 to 2018

Karn Vohra*, Eloise A. Marais*, William J. Bloss, Joel Schwartz, Loretta J. Mickley, Martin Van Damme, Lieven Clarisse, Pierre-F. Coheur

*Corresponding author. Email: e.marais@ucl.ac.uk (E.A.M.); k.vohra@ucl.ac.uk (K.V.)

Published 8 April 2022, *Sci. Adv.* **8**, eabm4435 (2022)
DOI: 10.1126/sciadv.abm4435

This PDF file includes:

Supplementary Text
Figs. S1 to S5
Tables S1 to S3
References

Supplementary Text

The following is supporting text for interpreting changes in premature mortality, estimating trends in precursor emissions that affect formation of surface ozone pollution, and to discern the relative contribution of traditional open burning of biomass and emerging anthropogenic sources to trends in air pollution. Trends in AOD (and thus $PM_{2.5}$) are reported in Supplementary Table 1, the change in premature mortality attributable to increase in urban exposure to $PM_{2.5}$ and the attributable fraction in each year is reported in Supplementary Table 2, and the change in per capita premature mortality is in Supplementary Table 3.

Trends in ozone production regimes

Satellite observations of tropospheric column ozone have limited sensitivity to surface concentrations of ozone *(99)*, so it is challenging to relate ozone concentrations in a single year from GEOS-Chem to the trends in satellite observations in the same way as we do for AOD and $PM_{2.5}$ (Material and Methods). Instead, we assess trends in sensitivity of surface ozone formation to precursor emissions (VOCs or NO_x) using ratios of formaldehyde (HCHO) to nitrogen dioxide (NO_2) ($HCHO/NO_2$) *(100,101)*. This aids policymakers in identifying ozone precursors (NO_x or VOCs) to target. The trends in these are in Supplementary Fig. 3B. These are determined with HCHO from all sources, rather than the HCHO from local sources, in order to relate the $HCHO/NO_2$ we obtain to values reported in the literature. Typically, $HCHO/NO_2 > \sim 2$ indicates that policies to mitigate ozone pollution should target NO_x sources, whereas $HCHO/NO_2 < 1$ supports targeting VOCs sources *(100)*. The transition between these regimes occurs for

$HCHO/NO_2$ values of 1-2 *(100-102)*. The actual threshold value for the NO_x-sensitive regime varies geographically, due to dependence on the local oxidation regime *(101,103-105)*. Of the tropical cities in Fig. 1, only Riyadh is in a NO_x-saturated regime ($HCHO/NO_2$ = 0.9) at the beginning and throughout the record, as values of NO_2 are large (~6 × 10^{15} molecules cm^{-2}) and the decline in NO_2 is slow and not significant (0.05 % a^{-1}; Fig. 2A). All other cities are in a NO_x-sensitive regime throughout the record. $HCHO/NO_2$ increases in Jakarta and Sana'a by 1.5 % a^{-1} due to a steep decline in NO_2 in Jakarta (Fig. 2A) and a steep increase in HCHO in Sana'a (Fig. 2C), so ozone formation in both cities remains NO_x-sensitive.

Contribution of trends in biogenic VOCs to trends in reactive NMVOCs

To assess if trends in biogenic VOCs influence trends in reactive NMVOCs, we select regions with minimal to no anthropogenic influence 50-100 km away from selected cities. We choose 5 cities spanning all regions of interest: Kinshasa and Abuja in Africa, Ahmedabad and Pune in South Asia and Phnom Penh in Southeast Asia. We also use Khartoum in Sudan as a control, as the surrounding rural areas are barren and so should have negligible biogenic VOC emissions. For each city, we sample HCHO columns after subtracting off the contribution from long-lived VOCs (Materials and Methods) over four 0.2° × 0.2° domains at least 50 km north-west, north-east, south-west and south-east of the city. We ignore domains influenced by anthropogenic sources such as nearby cities, clusters of urban settlements, major roads, airports, and industries identified with Google Maps. Trends are computed in all the data, in biomass burning months (values above 75th percentile) and non-biomass burning months (values below 75th percentile) (Materials and Methods). Using this approach, we find that HCHO trends in rural areas around the control (Khartoum) and the 5 test cities are not significant, supporting dominant influence of anthropogenic activity in trends in reactive NMVOCs (Fig. 4).

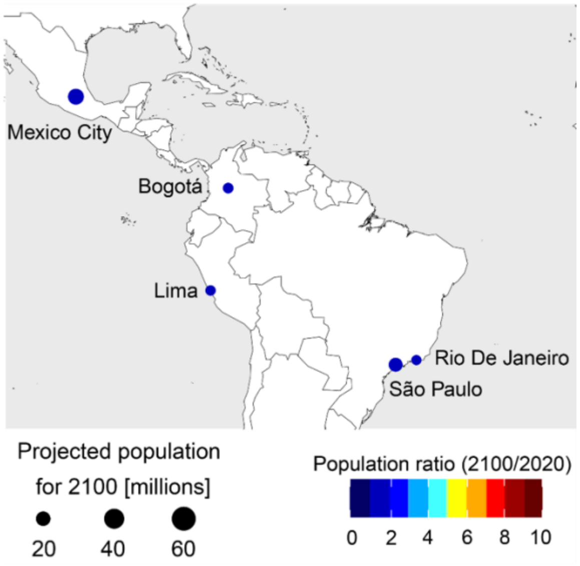

Supplementary Figure 1. Projected population growth for the tropical megacities in Central and South America. Circle sizes indicate projected 2100 population, and colors are 2100-to-2020 population ratios as indicators of population growth. Data for 2100 are from Hoornweg and Pope *(2)* and for 2020 from the UN *(1)*. Boxes discern cities in South Asia (red) and Southeast Asia (green).

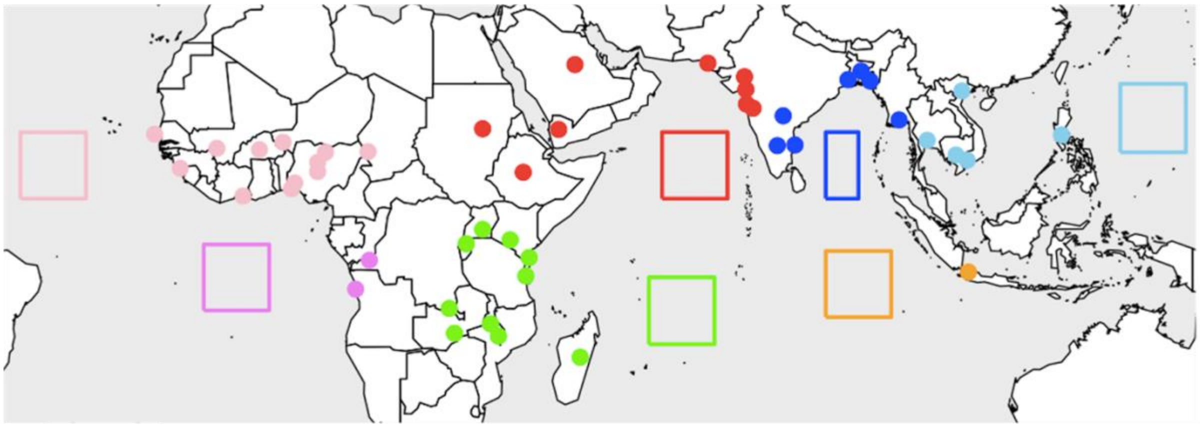

Supplementary Figure 2. Domains over remote oceans selected to calculate background HCHO from the Ozone Monitoring Instrument (OMI). Box colors indicate ocean domains used to determine background total column HCHO for cities with the same color (circles).

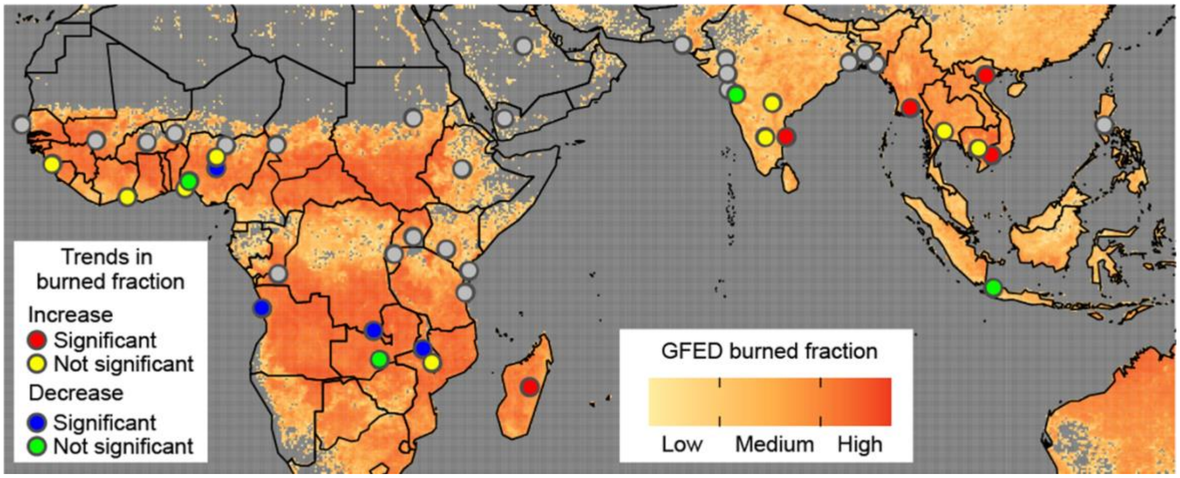

Supplementary Figure 3. Spatial distribution and trends in burned fraction in rapidly growing cities in the tropics. Background map is the multiyear (2005-2016) mean Global Fire Emissions Database version 4.1s (GFEDv4.1s) burned fraction at 0.25° × 0.25° for 2005-2016. The logarithmic scale distinguishes grid cells with low (<$10^{-7.5}$), medium ($10^{-7.5}$-$10^{-2.5}$), and high (>$10^{-2.5}$) burned fraction. Grey circles are cities with no discernible influence from biomass burning in our statistical analysis of the satellite observations (Materials and Methods).

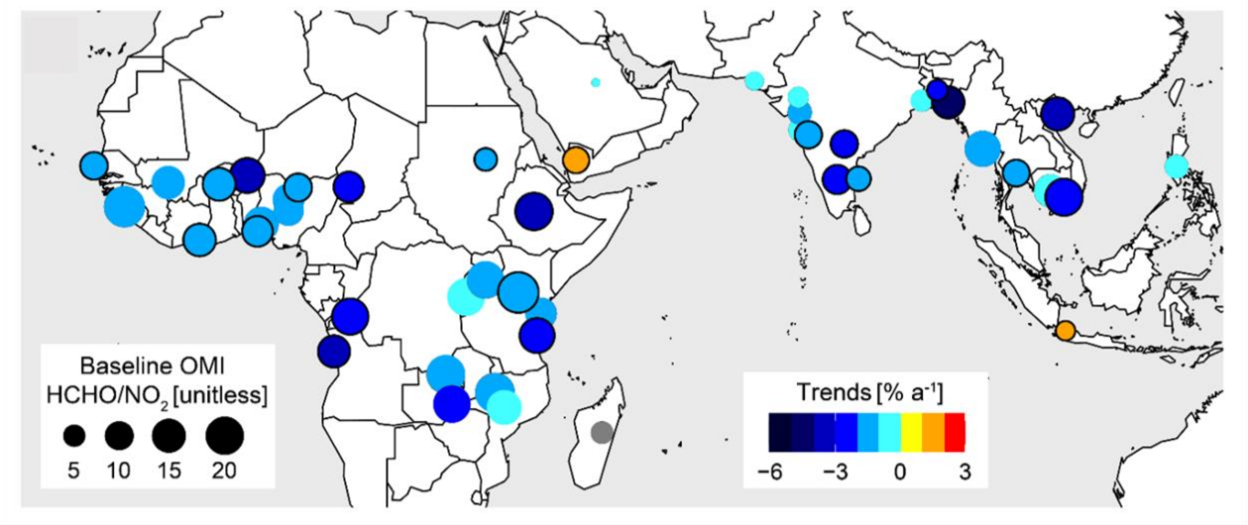

Supplementary Figure 4. Trends in proxy for ozone production regimes in rapidly growing cities in the tropics. Circle colors are relative trends in HCHO/NO_2 and sizes are values at the start of the record (baseline). Outlines identify significant trends at the 95 % CI. Warm colors indicate positive trends, cool colors negative trends. Cities with poor temporal coverage are grey.

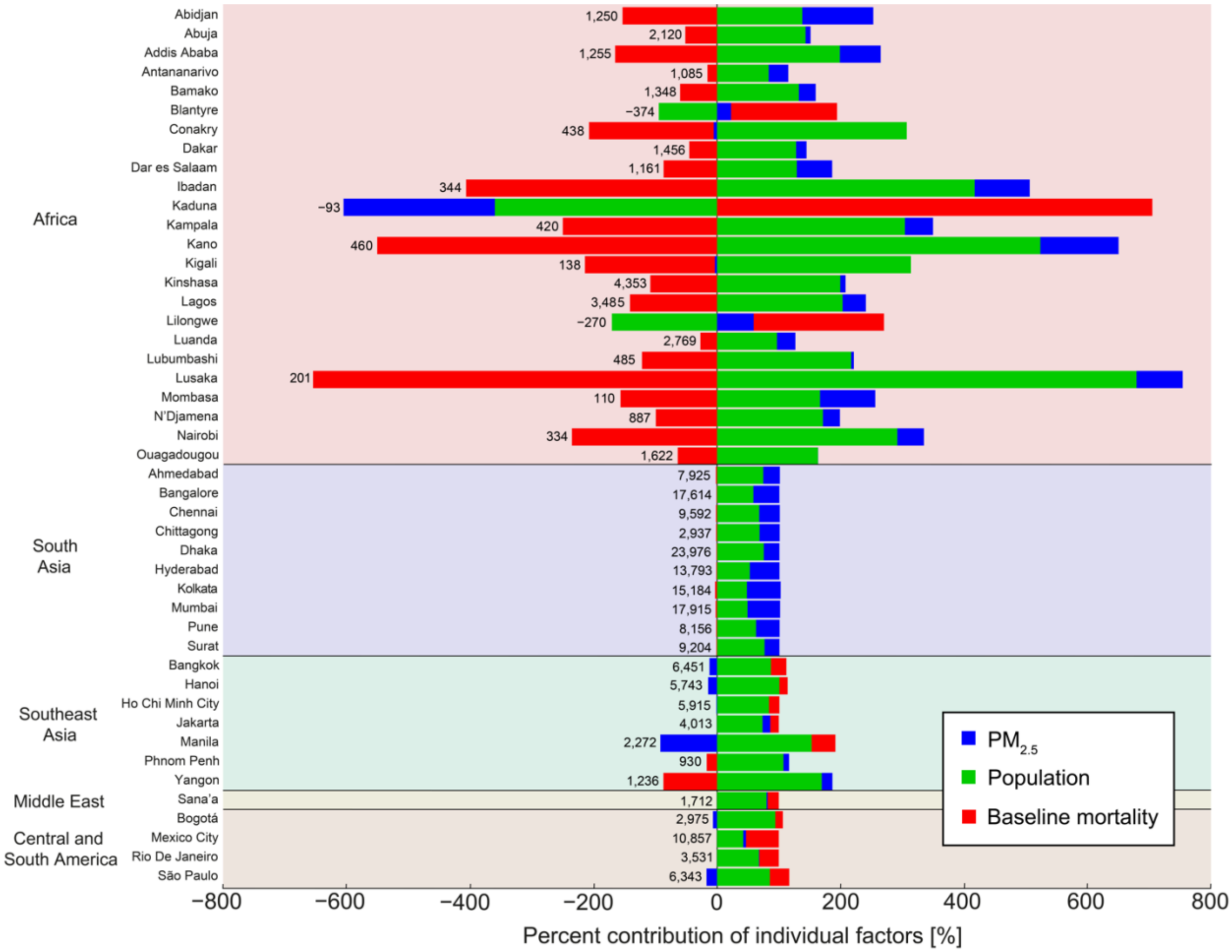

Supplementary Figure 5. Relative contribution of individual factors to changes in premature mortality from 2005 to 2018 in fast-growing tropical cities. Factors include changes in $PM_{2.5}$ (blue), population (green), and baseline mortality (red). Inset values are absolute changes in premature deaths from exposure to $PM_{2.5}$ for cities with discernible AOD trends (Fig. 3). The 95 % CI of these are in Supplementary Table 2.

Supplementary Table 1. Trends in air quality in the 51 fast-growing cities in the tropics.

City*	Long-term trends [% a^{-1}] †			
	NO_2	NH_3	Reactive NMVOCs	$PM_{2.5}$
**Africa**				
Abidjan§	**2.96** [1.3,5.1]	**5.07** [1.2,11.2]	**2.14** [0.8,3.7]	4.35 [-1.2,33.1]
Abuja	1.61[-0.1,3.9]	**3.72** [0.6,8.6]	0.39 [-0.6,1.6]	0.63 [-1.3,3.5]
Addis Ababa§	**6.35** [3.3,10.7]	1.22 [-0.5,3.3]	-0.96 [-3.5,3.6]	**3.27** [1.3,5.9]
Antananarivo§	**8.18** [4.3,14.1]	1.08 [-2.8,8.8]	—	2.95 [-0.7,9.6]
Bamako§	**4.10** [2.1, 6.7]	**5.08** [1.8,10.0]	2.51 [-0.9,8.0]	1.39 [-0.1,3.4]
Blantyre§	-0.86 [-3.2, 3.0]	-0.42 [-2.7,2.8]	0.25 [-2.1,4.0]	-1.06 [-3.2,2.4]
Conakry§	0.33 [-1.3,2.6]	1.27 [-0.9,4.3]	-0.68 [-1.8,0.7]	-0.09 [-1.0,0.9]
Dakar§	**3.64** [2.1,5.6]	**6.09** [1.4,14.4]	0.88 [-2.3,6.8]	0.61 [-1.3,4.1]
Dar es Salaam	**3.93** [1.6,7.1]	-0.94 [-3.9,4.1]	1.42 [-0.5,4.1]	**3.26** [0.9,6.6]
Ibadan	**3.02** [0.7,6.4]	**7.17** [2.6,14.9]	0.67 [-0.3,1.8]	0.91 [-2.3,7.5]
Kaduna§	**3.09** [1.4,5.2]	2.65 [-0.5,7.7]	0.36 [-2.5,4.6]	1.37 [-0.7,4.4]
Kampala§	**1.95** [0.2,4.3]	**2.79** [0.9,5.2]	-0.17 [-1.6,1.6]	1.19 [-0.6,3.6]
Kano§	**2.98** [1.9,4.3]	1.44 [-0.8,4.5]	**3.60** [0.8,8.0]	0.92 [-1.6,6.1]
Khartoum§	**1.77** [1.0,2.6]	**3.11** [0.4,6.8]	-0.83 [-3.9,4.8]	—
Kigali§	2.73 [-0.01,6.9]	**5.29** [2.0,10.1]	1.56 [-0.5,4.4]	-0.07 [-1.8,2.3]
**Kinshasa**§	**2.51** [0.4,5.4]	1.89 [-2.2,9.6]	**2.03** [0.7,3.6]	0.36 [-1.0,2.1]
**Lagos**§	**4.37** [2.3,7.2]	**8.31** [3.5,16.6]	**1.52** [0.5,2.8]	1.15 [-1.0,4.5]
Lilongwe§	-0.16 [-2.2,3.0]	**3.43** [0.7,7.3]	**3.63** [0.6,8.3]	-2.12 [-4.2,1.3]
Luanda	**7.36** [4.8,10.7]	**12.26** [2.8,40.8]	**3.90** [2.3,5.9]	**2.34** [0.6,4.6]
Lubumbashi	1.36 [-1.3,5.5]	**5.06** [0.6,13.0]	-0.28 [-1.6,1.4]	0.14 [-1.8,3.0]
Lusaka	0.74 [-1.1,3.4]	3.46 [-0.1,9.2]	-0.34 [-1.8,1.5]	0.80 [-1.4,4.1]
Mombasa§	-0.30 [-2.0,2.0]	2.95 [-1.7,12.2]	**-2.74** [-4.5,-0.1]	2.44 [-0.9,8.4]
N'Djamena§	**2.02** [0.9,3.4]	1.88 [-0.6,5.3]	-2.20 [-3.8,0.1]	1.12 [-0.5,4.3]
Nairobi	**1.81** [0.8,3.0]	**2.57** [1.0,4.5]	-0.71 [-1.7,0.4]	0.78 [-1.2,3.6]
Niamey§	**4.37** [2.8,6.3]	3.34 [-0.1,9.0]	-1.64 [-4.6,3.8]	—
Ouagadougou	**3.28** [2.0,4.8]	**4.76** [1.7,9.0]	**2.34** [0.3,5.2]	0.01 [-1.4,1.9]
**South Asia**				
Ahmedabad	**1.48** [0.7,2.3]	-1.27 [-3.0,1.0]	**1.75** [0.1,3.9]	2.25 [-0.1,5.6]
**Bangalore**	**3.80** [2.6,5.2]	-1.32 [-3.1,1.0]	0.61 [-1.1,2.9]	**7.77** [4.2,13.4]
**Chennai**	**2.33** [1.5,3.3]	**-2.61** [-4.3,-0.4]	**1.62** [0.02,3.7]	**3.32** [1.8,5.2]
Chittagong§	**14.07** [9.7,20.5]	-2.06 [-4.6,2.2]	0.59 [-2.0,4.6]	**2.72** [1.3,4.5]
**Dhaka**	**7.30** [5.1,10.2]	-0.62 [-3.0,2.9]	1.05 [-0.2,2.6]	**2.69** [1.4,4.3]
**Hyderabad**	**5.04** [3.5,7.0]	-0.76 [-2.4,1.3]	**2.67** [1.1,4.7]	**7.28** [5.1,10.1]
**Karachi**	**1.35** [0.7,2.1]	2.90 [-0.9,9.4]	**4.34** [0.4,11.9]	—

City*	Long-term trends [% a^{-1}] †			
	NO_2	NH_3	Reactive NMVOCs	$PM_{2.5}$
**Kolkata**	**1.12** [0.3,2.0]	**-3.75** [-5.2,-1.8]	1.31 [-0.001,2.9]	**2.49** [1.3,3.9]
**Mumbai**	**1.92** [1.1,2.9]	-1.32 [-2.8,0.5]	1.74 [-0.03,4.1]	**3.86** [2.5,5.5]
Pune	**3.90** [2.6,5.5]	0.33 [-1.8,3.2]	1.90 [-0.01,4.5]	**4.57** [2.4,7.5]
Surat	**0.80** [0.05,1.6]	-2.00 [-3.6,0.1]	**2.88** [1.2,5.0]	**3.73** [1.9,6.0]
Southeast Asia				
**Bangkok**	**1.44** [0.4,2.7]	**3.34** [1.0,6.5]	-0.67 [-1.3,0.1]	-0.75 [-1.6,0.2]
Hanoi	**7.73** [3.9,13.9]	**11.82** [5.0,25.4]	1.02 [-0.2,2.5]	-1.33 [-2.5,0.1]
Ho Chi Minh City	**4.35** [2.9,6.1]	**7.10** [4.4,10.6]	**1.74** [0.8,2.9]	-0.08 [-1.1,1.2]
**Jakarta**§	**-2.04** [-2.9,-1.1]	**3.87** [0.9,8.1]	**-1.68** [-2.4,-0.8]	0.49 [-0.7,2.0]
**Manila**§	**1.42** [0.2,2.9]	**2.64** [0.1,6.2]	1.23 [-0.9,4.4]	**-2.04** [-3.4.-0.3]
Phnom Penh§	1.21 [-0.2,3.0]	3.61 [-0.2,10.0]	**1.43** [0.05,3.2]	0.52 [-1.1,2.7]
Yangon	**4.64** [2.7,7.1]	-0.22 [-2.5,3.0]	**3.25** [1.1,6.1]	0.38 [-0.9,2.0]
Middle East				
Riyadh	-0.05 [-0.6,0.6]	**11.21** [1.6,44.4]	-0.81 [-4.1,6.6]	—
Sana'a	0.07 [-0.6,0.8]	1.15 [-3.0,9.3]	**10.62** [2.2,42.0]	0.19 [-1.1,1.7]
Central and South America				
**Bogotá**§	—	**7.28** [2.9,14.3]	2.92 [-2.3,19.8]	-0.32 [-3.1,5.4]
**Lima**	**4.44** [3.4,5.7]	**6.57** [4.5,9.2]	**1.35** [0.3,2.6]	—
**Mexico City**	0.43 [-0.4,1.3]	**2.00** [0.8,3.4]	**-1.39** [-2.1,-0.5]	0.28 [-0.9,1.8]
**Rio De Janeiro**	**1.26** [0.6,2.0]	0.28 [-1.9,3.4]	1.03 [-0.6,3.2]	0.02 [-1.6,2.2]
**São Paulo**	0.61 [-0.2,1.5]	**4.42** [1.0,9.6]	1.09 [-0.8,3.7]	-0.54 [-2.0,1.4]

* Current (2020) megacities are in bold. Small cities for which the satellite sampling domain is extended beyond the city boundary (Materials and Methods) are marked with §; † Trends in NO_2, reactive NMVOCs (HCHO) and $PM_{2.5}$ (AOD) are for 2005-2018 and trends in NH_3 are for 2008-2018. Trend values significant at the 95 % CI (given in square brackets) are in bold. Missing trends denoted with "—" are due to too few monthly means (Materials and Methods).

Supplementary Table 2. Premature deaths attributable to $PM_{2.5}$ exposure in the 51 fast-growing cities in the tropics.

City	2005 Premature deaths (1000s) [‡] [95 % CI]	2005 AF[‖] [%]	2018 Premature deaths (1000s) [‡] [95 % CI]	2018 AF[‖] [%]	2018 minus 2005[¶] Premature deaths (1000s) [‡] [95 % CI]	2018 minus 2005[¶] $PM_{2.5}$ [$\mu g\ m^{-3}$]
**Africa**						
Abidjan	**3.7** [2.1,5.2]	17	**4.9** [3.1,6.8]	23	1.3 [-1.2,3.7]	7.6
Abuja	**2.7** [0.4,5.0]	42	**4.8** [0.0,9.6]	44	2.1 [-3.2,7.5]	4.7
Addis Ababa	**3.3** [2.1,4.6]	24	**4.6** [2.7,6.4]	30	1.3 [-1.0,3.5]	9.5
Antananarivo	**0.7** [0.4,1.1]	10	**1.8** [1.0,2.6]	14	**1.1** [0.2,2.0]	2.7
Bamako	**3.0** [0.7, 5.2]	40	4.3 [-0.2,8.8]	45	1.3 [-3.7,6.4]	10.0
Blantyre	**1.0** [0.6, 1.4]	19	**0.7** [0.4,0.9]	17	-0.4[**] [-0.9,0.1]	-1.9
Conakry	**3.3** [1.2,5.5]	38	**3.8** [1.4,6.1]	38	0.4 [-2.7,3.6]	-0.6
Dakar	4.6 [-1.0,10.1]	47	6.0 [-2.8,14.9]	49	1.5 [-9.0,11.9]	5.5
Dar es Salaam	**1.4** [0.6,2.2]	9	**2.6** [1.3,3.9]	10	1.2 [-0.4,2.7]	2.1
Ibadan	**4.5** [1.9,7.2]	37	**4.9** [1.5,8.3]	39	0.3 [-4.0,4.7]	5.5
Kaduna	2.2 [-1.1,5.6]	49	2.2 [-3.4,7.7]	54	-0.1[**] [-6.6,6.4]	13.7
Kampala	**1.6** [0.9,2.2]	17	**2.0** [1.2,2.8]	19	0.4 [-0.6,1.5]	1.9
Kano	8.3 [-28.0,44.7]	59	8.8 [-69.3,86.9]	63	0.5 [-85.7,86.6]	12.1
Khartoum	—	—	—	—	—	—
Kigali	**0.8** [0.5,1.1]	22	**0.9** [0.6,1.3]	22	0.1 [-0.3,0.6]	-0.2
Kinshasa	**13.3** [7.4,19.2]	32	**17.7** [9.6,25.8]	32	4.4 [-5.7,14.4]	1.6
Lagos	**14.9** [7.4,22.4]	34	**18.4** [7.4,29.3]	37	3.5 [-9.8,16.7]	6.1
Lilongwe	**0.9** [0.5,1.3]	17	**0.6** [0.3,0.9]	13	-0.3[**] [-0.7,0.2]	-2.9
Luanda	**2.7** [1.5,3.9]	14	**5.4** [3.2,7.6]	18	**2.8** [0.2,5.3]	3.2
Lubumbashi	**1.7** [1.1,2.3]	23	**2.2** [1.4,3.0]	23	0.5 [-0.5,1.5]	0.3
Lusaka	**1.9** [1.1,2.7]	16	**2.1** [1.2,3.0]	17	0.2 [-1.0,1.4]	1.2
Mombasa	**0.3** [0.1,0.5]	6	**0.4** [0.2,0.7]	8	0.1 [-1.0,1.4]	1.2
N'Djamena	2.5 [-3.2,8.1]	53	3.4 [-10.5,17.3]	58	0.9 [-14.1,15.9]	12.7
Nairobi	**1.5** [0.7,2.3]	8	**1.8** [0.9,2.8]	9	0.3 [-0.9,1.6]	0.5
Niamey	—	—	—	—	—	—
Ouagadougou	**3.1** [0.5,5.6]	42	**4.7** [0.8,8.6]	42	1.6 [-3.0,6.2]	0.1
**South Asia**						
Ahmedabad	**10.5** [5.3,15.7]	34	**18.4** [5.4,31.4]	40	7.9 [-6.1,21.9]	12.2
Bangalore	**9.5** [5.9,13.1]	25	**27.1** [8.9,45.3]	39	17.6 [-0.9,36.1]	27.5
Chennai	**11.2** [6.9,15.5]	27	**20.8** [11.1,30.6]	33	9.6 [-1.1,20.2]	11.8
Chittagong	**4.2** [2.6,5.9]	27	**7.2** [4.0,10.4]	32	2.9 [-0.7,6.6]	9.6
Dhaka	**21.2** [5.1,37.3]	41	45.2 [-22.1,112.4]	49	24.0 [-45.2,93.1]	20.2

City	2005 Premature deaths (1000s) ‡ [95 % CI]	2005 AF‖ [%]	2018 Premature deaths (1000s) ‡ [95 % CI]	2018 AF‖ [%]	2018 minus 2005¶ Premature deaths (1000s) ‡ [95 % CI]	2018 minus 2005¶ $PM_{2.5}$ [$\mu g\ m^{-3}$]
Hyderabad	**9.9** [6.1,13.8]	27	**23.7** [4.8,42.6]	41	13.8 [-5.5,33.1]	29.4
Karachi	—	—	—	—	—	—
Kolkata	39.2 [-32.5,110.8]	51	54.4 [-325.4,434.1]	61	15.2 [-371.3,401.7]	27.6
Mumbai	**30.4** [17.1,43.8]	31	**48.3** [12.9,83.8]	40	17.9 [-20.0,55.8]	18.8
Pune	**7.4** [4.2,10.5]	31	**15.5** [3.5,27.5]	41	8.2 [-4.2,20.6]	21.7
Surat	**5.8** [3.4,8.1]	29	**15.0** [6.1,23.9]	37	**9.2** [0.0,18.4]	16.0
Southeast Asia						
Bangkok	**11.8** [7.1,16.5]	28	**18.3** [11.2,25.3]	27	6.5 [-2.0,14.9]	-2.6
Hanoi	5.2 [-0.4,10.8]	45	**10.9** [2.6,19.3]	41	5.7 [-4.3,15.8]	-10.6
Ho Chi Minh City	**7.3** [4.5,10.1]	26	**13.2** [8.2,18.2]	26	**5.9** [0.2,11.7]	-0.3
Jakarta	**12.5** [7.8,17.2]	25	**16.5** [10.2,22.8]	26	4.0 [-3.8,11.9]	1.4
Manila	**9.8** [6.1,13.5]	21	**12.0** [7.1,17.0]	17	2.3 [-3.9,8.5]	-4.0
Phnom Penh	**1.6** [1.0,2.2]	21	**2.5** [1.6,3.5]	22	0.9 [-0.2,2.1]	1.1
Yangon	**5.7** [3.5,8.0]	19	**7.0** [4.2,9.7]	20	1.2 [-2.3,4.8]	0.7
Middle East						
Riyadh	—	—	—	—	—	—
Sana'a	**1.6** [1.0,2.2]	26	**3.3** [2.0,4.5]	26	**1.7** [0.3,3.1]	0.6
Central and South America						
Bogotá	**4.7** [2.7,6.8]	16	**7.7** [4.3,11.1]	15	3.0 [-1.0,6.9]	-0.4
Lima	—	—	—	—	—	—
Mexico City	**20.3** [12.6,28.0]	26	**31.1** [19.2,43.0]	27	10.9 [-3.3,25.0]	0.9
Rio De Janeiro	**10.8** [6.3,15.2]	17	**14.3** [8.4,20.2]	18	3.5 [-3.8,10.9]	0.03
São Paulo	**19.3** [11.8,26.7]	20	**25.6** [15.5,35.7]	19	6.3 [-6.2,18.9]	-1.1

¶ Accounts for temporal changes in population and baseline mortality. ‡ Premature mortality values significant at the 95 % CI are in bold. Missing data denoted with "—" are for cities with no discernible trends in $PM_{2.5}$ (Fig. 3). ‖ AF: Fraction of deaths attributable to ambient $PM_{2.5}$ exposure for the population > 14 years old. ** The decline in premature mortality in Kaduna is due to a decline in total premature deaths, and in Blantyre and Lilongwe is due to declines in both $PM_{2.5}$ and total premature deaths.

Supplementary Table 3. Per capita premature mortality attributable to $PM_{2.5}$ exposure in the 51 fast-growing cities in the tropics.

City	Per capita premature mortality (per 10^5 people) ‡ [95 % CI]		
	2005	2018	2018 minus 2005
**Africa**			
Abidjan	**106.7** [61.7,151.7]	**99.2** [62.1,136.3]	-7.5 [-65.8,50.8]
Abuja	**206.4** [30.9,382.0]	**160.4** [1.0,319.7]	-46.1 [-283.2,191.1]
Addis Ababa	**125.9** [78.8,172.9]	**101.4** [59.8,143.1]	-24.4 [-87.2,38.4]
Antananarivo	**46.5** [22.8,70.1]	**57.9** [31.3,84.4]	11.4 [-24.1,47.0]
Bamako	**200.7** [49.9, 351.6]	175.6 [-7.5,358.7]	-25.2 [-262.4,212.1]
Blantyre	**169.7** [102.2, 237.2]	**74.1** [43.2,105.1]	**-95.5** [-169.8,-21.3]
Conakry	**248.6** [91.0,406.3]	**203.4** [76.6,330.2]	-45.2 [-247.6,157.1]
Dakar	213.7 [-46.0,473.5]	200.2 [-93.7,494.1]	-13.5 [-405.8,378.7]
Dar es Salaam	**49.3** [22.1,76.4]	**42.0** [20.7,63.3]	-7.3 [-41.8,27.2]
Ibadan	**181.0** [74.6,287.4]	**143.1** [43.3,243.0]	-37.9 [-183.8,108.0]
Kaduna	241.1 [-120.2,602.3]	198.0 [-309.4,705.3]	-43.1 [-665.9,579.7]
Kampala	**99.9** [57.9,141.9]	**65.0** [39.1,91.0]	-34.9 [-84.3,14.5]
Kano	287.1 [-968.7,1543.0]	227.8 [-1800.4,2255.9]	-59.4 [-2444.9,2326.1]
Khartoum	—	—	—
Kigali	**116.3** [72.6,160.0]	**85.4** [53.3,117.6]	-30.9 [-85.1,23.4]
Kinshasa	**175.4** [97.5,253.2]	**131.4** [71.1,191.7]	-44.0 [-142.5,54.5]
Lagos	**168.1** [83.8,252.4]	**134.8** [54.6,215.0]	-33.3 [-149.7,83.1]
Lilongwe	**149.6** [86.4,212.8]	**58.3** [31.1,85.4]	**-91.3** [-160.1,-22.5]
Luanda	**68.5** [37.6,99.5]	**70.1** [41.4,98.8]	1.6 [-40.6,43.8]
Lubumbashi	**126.7** [79.3,174.1]	**93.7** [58.7,128.7]	-33.0 [-92.0,25.9]
Lusaka	**140.4** [79.9,200.8]	**81.5** [47.6,115.3]	-58.9 [-128.1,10.4]
Mombasa	**39.0** [16.5,61.4]	**34.3** [15.4,53.2]	-4.7 [-34.0,24.7]
N'Djamena	299.9 [-384.3,984.2]	250.6 [-784.9,1286.1]	-49.3 [-1290.5,1191.9]
Nairobi	**56.3** [26.0,86.7]	**41.3** [19.5,63.1]	-15.0 [-52.4,22.3]
Niamey	—	—	—
Ouagadougou	**230.3** [40.2,420.4]	**180.9** [31.2,330.6]	-49.4 [-291.4,192.6]
**South Asia**			
Ahmedabad	**190.9** [96.5,285.3]	**238.6** [70.0,407.2]	47.7 [-145.5,240.9]
Bangalore	**140.2** [87.6,192.9]	**234.1** [77.2,391.0]	93.9 [-71.6,259.4]
Chennai	**150.3** [92.6,207.9]	**198.3** [105.5,291.0]	48.0 [-61.2,157.2]
Chittagong	**114.2** [70.3,158.2]	**148.2** [81.6,214.8]	34.0 [-45.8,113.8]
Dhaka	**171.9** [41.7,302.1]	227.6 [-111.2,566.4]	55.7 [-307.3,418.6]
Hyderabad	**152.2** [93.4,210.9]	**248.6** [50.3,446.9]	96.4 [-110.4,303.3]

City	Per capita premature mortality (per 10^5 people) ‡ [95 % CI]		
	2005	2018	2018 minus 2005
Karachi	—	—	—
Kolkata	288.2 [-239.0,815.3]	370.2 [-2216.3,2956.8]	82.1 [-2557.7,2721.8]
Mumbai	**176.2** [98.9,253.5]	**241.7** [64.4,419.1]	65.5 [-128.0,259.0]
Pune	**172.4** [98.7,246.0]	**246.0** [56.1,435.9]	73.6 [-130.1,277.3]
Surat	**165.2** [97.6,232.9]	**223.6** [90.7,356.5]	58.4 [-90.8,207.5]
**Southeast Asia**			
Bangkok	**162.3** [97.6,226.9]	**180.6** [111.1,250.2]	18.4 [-76.6,113.3]
Hanoi	240.7 [-19.0,500.5]	**252.0** [59.9,444.1]	11.3 [-311.8,334.3]
Ho Chi Minh City	**140.0** [86.7,193.3]	**162.1** [100.5,223.7]	22.1 [-59.3,103.6]
Jakarta	**139.1** [86.8,191.4]	**156.8** [97.2,216.3]	17.7 [-61.6,97.0]
Manila	**90.9** [56.3,125.5]	**89.2** [52.5,126.0]	-1.7 [-52.1,48.8]
Phnom Penh	**120.7** [74.8,166.6]	**127.5** [79.5,175.4]	6.8 [-59.6,73.1]
Yangon	**145.0** [87.4,202.5]	**135.4** [82.4,188.4]	-9.6 [-87.8,68.6]
**Middle East**			
Riyadh	—	—	—
Sana’a	**90.6** [56.0,124.6]	**116.9** [72.3,161.5]	26.6 [-29.6,82.9]
**Central and South America**			
Bogotá	**64.7** [36.7,92.8]	**73.4** [41.1,05.7]	8.7 [-34.1,51.4]
Lima	—	—	—
Mexico City	**105.2** [65.3,145.1]	**145.1** [89.6,200.6]	40.0 [-28.4,108.3]
Rio De Janeiro	**90.9** [53.5,128.3]	**107.9** [63.5,152.3]	17.0 [-41.1,75.1]
São Paulo	**105.4** [64.7,146.0]	**118.9** [72.165.7]	13.5 [-48.4,75.5]

‡ Per capita mortality values significant at the 95 % CI are in bold. Missing data denoted with “—” are for cities with no discernible trends in $PM_{2.5}$ (Fig. 3).

REFERENCES AND NOTES

1. UN, 2018. World Urbanization Prospects: The 2018 revision, online edition; https://population.un.org/wup/Download/ [accessed 14 June 2021].
2. D. Hoornweg, K. Pope, Population predictions for the world's largest cities in the 21st century. Environ. Urban. 29, 195–216 (2017).
3. R. M. Duren, C. E. Miller, Measuring the carbon emissions of megacities. Nat. Clim. Change 2, 560–562 (2012).
4. M. Krzyzanowski, J. S. Apte, S. P. Bonjour, M. Brauer, A. Cohen, A. M. Prüss-Ustun, Air pollution in the mega-cities. Curr. Envir. Health Rpt. 1, 185–191 (2014).
5. S. C. Fang, E. G. Rodrigues, D. C. Christiani, Environmental health hazards in the tropics, in Hunter's Tropical Medicine and Emerging Infectious Diseases, E. T. Ryan, D. R. Hill, T. Solomon, N. E. Aronson, T. P. Endy, Eds. (Elsevier, ed. 10, 2020), pp. 200–208.
6. K. Vohra, E. A. Marais, S. Suckra, L. Kramer, W. J. Bloss, R. Sahu, A. Gaur, S. N. Tripathi, M. Van Damme, L. Clarisse, P. F. Coheur, Long-term trends in air quality in major cities in the UK and India: A view from space. Atmos. Chem. Phys. 21, 6275–6296 (2021).
7. S. Yin, X. F. Wang, X. R. Zhang, M. Guo, M. Miura, Y. Xiao, Influence of biomass burning on local air pollution in mainland Southeast Asia from 2001 to 2016. Environ. Pollut. 254, 112949 (2019).
8. A. M. Aghedo, M. G. Schultz, S. Rast, The influence of African air pollution on regional and global tropospheric ozone. Atmos. Chem. Phys. 7, 1193–1212 (2007).
9. C. L. Reddington, L. Conibear, C. Knote, B. Silver, Y. J. Li, C. K. Chan, S. R. Arnold, D. V. Spracklen, Exploring the impacts of anthropogenic emission sectors on PM2.5 and human health in South and East Asia. Atmos. Chem. Phys. 19, 11887–11910 (2019).
10. E. A. Marais, C. Wiedinmyer, Air quality impact of diffuse and inefficient combustion emissions in Africa (DICE-Africa). Environ. Sci. Technol. 50, 10739–10745 (2016).
11. S. E. Bauer, U. Im, K. Mezuman, C. Y. Gao, Desert dust, industrialization, and agricultural fires: Health impacts of outdoor air pollution in Africa. J. Geophys. Res. Atmos. 124, 4104–4120 (2019).
12. A. S. Bockarie, E. A. Marais, A. R. MacKenzie, Air pollution and climate forcing of the charcoal industry in Africa. Environ. Sci. Technol. 54, 13429–13438 (2020).
13. E. A. Marais, D. J. Jacob, K. Wecht, C. Lerot, L. Zhang, K. Yu, T. P. Kurosu, K. Chance, B. Sauvage, Anthropogenic emissions in Nigeria and implications for atmospheric ozone pollution: A view from space. Atmos. Environ. 99, 32–40 (2014).
14. N. Ojha, A. Sharma, M. Kumar, I. Girach, T. U. Ansari, S. K. Sharma, N. Singh, A. Pozzer, S. S. Gunthe, On the widespread enhancement in fine particulate matter across the Indo-Gangetic Plain towards winter. Sci. Rep. 10, 5862 (2020).
15. A. M. Thompson, W. K. Tao, K. E. Pickering, J. R. Scala, J. Simpson, Tropical deep convection and ozone formation. B. Am. Meteorol. Soc. 78, 1043–1054 (1997).
16. J. E. Hickman, N. Andela, K. Tsigaridis, C. Galy-Lacaux, M. Ossohou, S. E. Bauer, Reductions in NO2 burden over north equatorial Africa from decline in biomass burning in spite of growing fossil fuel use, 2005 to 2017. Proc. Natl. Acad. Sci. U.S.A. 118, e2002579 (2021).
17. P. Bhardwaj, M. Naja, R. Kumar, H. C. Chandola, Seasonal, interannual, and long-term variabilities in biomass burning activity over South Asia. Environ. Sci. Pollut. R. 23, 4397–4410 (2016).

18. GBD 2017 Risk Factor Collaborators, Global, regional, and national comparative risk assessment of 84 behavioural, environmental and occupational, and metabolic risks or clusters of risks for 195 countries and territories, 1990–2017: A systematic analysis for the Global Burden of Disease Study 2017. Lancet 392, 1923–1994 (2018).
19. K. Vohra, A. Vodonos, J. Schwartz, E. A. Marais, M. P. Sulprizio, L. J. Mickley, Global mortality from outdoor fine particle pollution generated by fossil fuel combustion: Results from GEOS-Chem. Environ. Res. 195, 110754 (2021).
20. Health Effects Institute, State of Global Air 2020. Special Report (Health Effects Institute, 2020).
21. World Bank, World Development Indicators (2019); https://databank.worldbank.org/source/world-development-indicators [accessed 14 June 2021].
22. R. V. Martin, M. Brauer, A. van Donkelaar, G. Shaddick, U. Narain, S. Dey, No one knows which city has the highest concentration of fine particulate matter. Atmos. Environ. X 3, 100040 (2019).
23. M. Brauer, S. K. Guttikunda, K. Nishad, S. Dey, S. N. Tripathi, C. Weagle, R. V. Martin, Examination of monitoring approaches for ambient air pollution: A case study for India. Atmos. Environ. 216, 116940 (2019).
24. R. Sahu, A. Nagal, K. K. Dixit, H. Unnibhavi, S. Mantravadi, S. Nair, Y. Simmhan, B. Mishra, R. Zele, R. Sutaria, V. M. Motghare, P. Kar, S. N. Tripathi, Robust statistical calibration and characterization of portable low-cost air quality monitoring sensors to quantify real-time O3 and NO2 concentrations in diverse environments. Atmos. Meas. Tech. 14, 37–52 (2021).
25. B. N. Duncan, L. N. Lamsal, A. M. Thompson, Y. Yoshida, Z. F. Lu, D. G. Streets, M. M. Hurwitz, K. E. Pickering, A space-based, high-resolution view of notable changes in urban NOx pollution around the world (2005–2014). J. Geophys. Res. Atmos. 121, 976–996 (2016).
26. M. Van Damme, L. Clarisse, B. Franco, M. A. Sutton, J. W. Erisman, R. W. Kruit, M. van Zanten, S. Whitburn, J. Hadji-Lazaro, D. Hurtmans, C. Clerbaux, P. F. Coheur, Global, regional and national trends of atmospheric ammonia derived from a decadal (2008–2018) satellite record. Environ. Res. Lett., 005017 (2021).
27. A. Hilboll, A. Richter, J. P. Burrows, Long-term changes of tropospheric NO2 over megacities derived from multiple satellite instruments. Atmos. Chem. Phys. 13, 4145–4169 (2013).
28. P. Alpert, O. Shvainshtein, P. Kishcha, AOD trends over megacities based on space monitoring using MODIS and MISR. Am. J. Clim. Change 01, 117–131 (2012).
29. I. De Smedt, T. Stavrakou, J. F. Muller, R. J. van der A, M. Van Roozendael, Trend detection in satellite observations of formaldehyde tropospheric columns. Geophys. Res. Lett. 37, L18808 (2010).
30. E. A. Marais, D. J. Jacob, T. P. Kurosu, K. Chance, J. G. Murphy, C. Reeves, G. Mills, S. Casadio, D. B. Millet, M. P. Barkley, F. Paulot, J. Mao, Isoprene emissions in Africa inferred from OMI observations of formaldehyde columns. Atmos. Chem. Phys. 12, 6219–6235 (2012).
31. P. Lalitaporn, G. Kurata, Y. Matsuoka, N. Thongboonchoo, V. Surapipith, Long-term analysis of NO2, CO, and AOD seasonal variability using satellite observations over Asia

and intercomparison with emission inventories and model. Air Qual. Atmos. Health 6, 655–672 (2013).
32. S. D. A. Kusumaningtyas, E. Aldrian, T. Wati, D. Atmoko, S. Sunaryo, The recent state of ambient air quality in Jakarta. Aerosol Air Qual. Res. 18, 2343–2354 (2018).
33. P. Schneider, W. A. Lahoz, R. van der A, Recent satellite-based trends of tropospheric nitrogen dioxide over large urban agglomerations worldwide. Atmos. Chem. Phys. 15, 1205–1220 (2015).
34. A. K. Georgoulias, R. J. van der A, P. Stammes, K. F. Boersma, H. J. Eskes, Trends and trend reversal detection in 2 decades of tropospheric NO2 satellite observations. Atmos. Chem. Phys. 19, 6269–6294 (2019).
35. J. Lelieveld, S. Beirle, C. Hormann, G. Stenchikov, T. Wagner, Abrupt recent trend changes in atmospheric nitrogen dioxide over the Middle East. Sci. Adv. 1, e1500498 (2015).
36. L. Clarisse, M. Van Damme, C. Clerbaux, P. F. Coheur, Tracking down global NH3 point sources with wind-adjusted superresolution. Atmos. Meas. Tech. 12, 5457–5473 (2019).
37. M. Van Damme, L. Clarisse, S. Whitburn, J. Hadji-Lazaro, D. Hurtmans, C. Clerbaux, P. F. Coheur, Industrial and agricultural ammonia point sources exposed. Nature 564, 99–103 (2018).
38. C. Li, R. V. Martin, A. van Donkelaar, B. L. Boys, M. S. Hammer, J. W. Xu, E. A. Marais, A. Reff, M. Strum, D. A. Ridley, M. Crippa, M. Brauer, Q. Zhang, Trends in chemical composition of global and regional population-weighted fine particulate matter estimated for 25 years. Environ. Sci. Technol. 51, 11185–11195 (2017).
39. S. Provencal, P. Kishcha, A. M. da Silva, E. Elhacham, P. Alpert, AOD distributions and trends of major aerosol species over a selection of the world's most populated cities based on the 1st version of NASA's MERRA Aerosol Reanalysis. Urban Clim. 20, 168–191 (2017).
40. T. Wang, Y. Song, Z. Xu, M. Liu, T. Xu, W. Liao, L. Yin, X. Cai, L. Kang, H. Zhang, T. Zhu, Why is the Indo-Gangetic Plain the region with the largest NH3 column in the globe during pre-monsoon and monsoon seasons? Atmos. Chem. Phys. 20, 8727–8736 (2020).
41. C. Li, C. McLinden, V. Fioletov, N. Krotkov, S. Carn, J. Joiner, D. Streets, H. He, X. R. Ren, Z. Q. Li, R. R. Dickerson, India is overtaking China as the world's largest emitter of anthropogenic sulfur dioxide. Sci. Rep. 7, 14304 (2017).
42. S. S. Gunthe, P. F. Liu, U. Panda, S. S. Raj, A. Sharma, E. Darbyshire, E. Reyes-Villegas, J. Allan, Y. Chen, X. Wang, S. J. Song, M. L. Pohlker, L. H. Shi, Y. Wang, S. M. Kommula, T. J. Liu, R. Ravikrishna, G. McFiggans, L. J. Mickley, S. T. Martin, U. Poschl, M. O. Andreae, H. Coe, Enhanced aerosol particle growth sustained by high continental chlorine emission in India. Nat. Geosci. 14, 77–84 (2021).
43. D. Hoornweg, P. Bhada-Tata, C. Kennedy, Environment: Waste production must peak this century. Nature 502, 615–617 (2013).
44. R. T. Xu, S. F. Pan, J. Chen, G. S. Chen, J. Yang, S. R. S. Dangal, J. P. Shepard, H. Q. Tian, Half-century ammonia emissions from agricultural systems in Southern Asia: Magnitude, spatiotemporal patterns, and implications for human health. Geohealth 2, 40–53 (2018).
45. S. K. Sharma, G. Kotnala, T. K. Mandal, Spatial variability and sources of atmospheric ammonia in India: A review. Aerosol Sci. Eng. 4, 1–8 (2020).
46. J. Kuttippurath, A. Singh, S. P. Dash, N. Mallick, C. Clerbaux, M. Van Damme, L. Clarisse, P. F. Coheur, S. Raj, K. Abbhishek, H. Varikoden, Record high levels of atmospheric ammonia over India: Spatial and temporal analyses. Sci. Total Environ. 740, 139986 (2020).

47. N. Damanik, H. C. Ong, C. W. Tong, T. M. I. Mahlia, A. S. Silitonga, A review on the engine performance and exhaust emission characteristics of diesel engines fueled with biodiesel blends. Environ. Sci. Pollut. R. 25, 15307–15325 (2018).
48. Statista, Biodiesel consumption in Indonesia 2010–2020 (2021); www.statista.com/statistics/1055635/indonesia-biodiesel-consumption/ [accessed 25 June 2021].
49. P. Sicard, A. Anav, A. De Marco, E. Paoletti, Projected global ground-level ozone impacts on vegetation under different emission and climate scenarios. Atmos. Chem. Phys. 17, 12177–12196 (2017).
50. F. Hayes, H. Harmens, K. Sharps, A. Radbourne, Ozone dose-response relationships for tropical crops reveal potential threat to legume and wheat production, but not to millets. Sci. Afr. 9, e00482 (2020).
51. B. C. McDonald, J. A. de Gouw, J. B. Gilman, S. H. Jathar, A. Akherati, C. D. Cappa, J. L. Jimenez, J. Lee-Taylor, P. L. Hayes, S. A. McKeen, Y. Y. Cui, S. W. Kim, D. R. Gentner, G. Isaacman-VanWertz, A. H. Goldstein, R. A. Harley, G. J. Frost, J. M. Roberts, T. B. Ryerson, M. Trainer, Volatile chemical products emerging as largest petrochemical source of urban organic emissions. Science 359, 760–764 (2018).
52. P. Gupta, S. A. Christopher, J. Wang, R. Gehrig, Y. Lee, N. Kumar, Satellite remote sensing of particulate matter and air quality assessment over global cities. Atmos. Environ. 40, 5880–5892 (2006).
53. A. van Donkelaar, R. V. Martin, M. Brauer, B. L. Boys, Use of satellite observations for long-term exposure assessment of global concentrations of fine particulate matter. Environ. Health Persp. 123, 135–143 (2015).
54. A. van Donkelaar, R. V. Martin, M. Brauer, N. C. Hsu, R. A. Kahn, R. C. Levy, A. Lyapustin, A. M. Sayer, D. M. Winker, Global estimates of fine particulate matter using a combined geophysical-statistical method with information from satellites, models, and monitors. Environ. Sci. Technol. 50, 3762–3772 (2016).
55. G. Shaddick, M. L. Thomas, H. Amini, D. Broday, A. Cohen, J. Frostad, A. Green, S. Gumy, Y. Liu, R. V. Martin, A. Pruss-Ustun, D. Simpson, A. van Donkelaar, M. Brauer, Data integration for the assessment of population exposure to ambient air pollution for Global Burden of Disease assessment. Environ. Sci. Technol. 52, 9069–9078 (2018).
56. A. Farahat, Comparative analysis of MODIS, MISR, and AERONET climatology over the Middle East and North Africa. Ann. Geophys. 37, 49–64 (2019).
57. M. S. Hammer, A. van Donkelaar, C. Li, A. Lyapustin, A. M. Sayer, N. C. Hsu, R. C. Levy, M. J. Garay, O. V. Kalashnikova, R. A. Kahn, M. Brauer, J. S. Apte, D. K. Henze, L. Zhang, Q. Zhang, B. Ford, J. R. Pierce, R. V. Martin, Global estimates and long-term trends of fine particulate matter concentrations (1998–2018). Environ. Sci. Technol. 54, 7879–7890 (2020).
58. Z. Hu, Q. Jin, Y. Ma, B. Pu, Z. Ji, Y. Wang, W. Dong, Temporal evolution of aerosols and their extreme events in polluted Asian regions during Terra's 20-year observations. Remote Sens. Environ. 263, 112541 (2021).
59. S. A. Logothetis, V. Salamalikis, A. Gkikas, S. Kazadzis, V. Amiridis, A. Kazantzidis, 15-year variability of desert dust optical depth on global and regional scales. Atmos. Chem. Phys. 21, 16499–16529 (2021).
60. M. Santoso, D. D. Lestiani, E. Damastuti, S. Kurniawati, I. Kusmartini, D. P. D. Atmodjo, D. K. Sari, T. Muhtarom, D. A. Permadi, P. K. Hopke, Long term characteristics of

atmospheric particulate matter and compositions in Jakarta, Indonesia. Atmos. Pollut. Res. 11, 2215–2225 (2020).
61. S. N. Koplitz, D. J. Jacob, M. P. Sulprizio, L. Myllyvirta, C. Reid, Burden of disease from rising coal-fired power plant emissions in Southeast Asia. Environ. Sci. Technol. 51, 1467–1476 (2017).
62. R. R. Buchholz, H. M. Worden, M. Park, G. Francis, M. N. Deeter, D. P. Edwards, L. K. Emmons, B. Gaubert, J. Gille, S. Martinez-Alonso, W. F. Tang, R. Kumar, J. R. Drummond, C. Clerbaux, M. George, P. F. Coheur, D. Hurtmans, K. W. Bowman, M. Luo, V. H. Payne, J. R. Worden, M. Chin, R. C. Levy, J. Warner, Z. G. Wei, S. S. Kulawik, Air pollution trends measured from Terra: CO and AOD over industrial, fire-prone, and background regions. Remote Sens. Environ. 256, 112275 (2021).
63. F. K. Dwomoh, M. C. Wimberly, Fire regimes and forest resilience: Alternative vegetation states in the West African tropics. Landsc. Ecol. 32, 1849–1865 (2017).
64. E. E. McDuffie, R. V. Martin, J. V. Spadaro, R. Burnett, S. J. Smith, P. O'Rourke, M. S. Hammer, A. van Donkelaar, L. Bindle, V. Shah, L. Jaeglé, G. Luo, F. Yu, J. A. Adeniran, J. Lin, M. Brauer, Source sector and fuel contributions to ambient PM2.5 and attributable mortality across multiple spatial scales. Nat. Commun. 12, 3594 (2021).
65. J. L. Laughner, R. C. Cohen, Direct observation of changing NOx lifetime in North American cities. Science 366, 723–727 (2019).
66. R. F. Silvern, D. J. Jacob, L. J. Mickley, M. P. Sulprizio, K. R. Travis, E. A. Marais, R. C. Cohen, J. L. Laughner, S. Choi, J. Joiner, L. N. Lamsal, Using satellite observations of tropospheric NO2 columns to infer long-term trends in US NOx emissions: The importance of accounting for the free tropospheric NO2 background. Atmos. Chem. Phys. 19, 8863–8878 (2019).
67. Y. Z. Zhang, D. J. Jacob, X. Lu, J. D. Maasakkers, T. R. Scarpelli, J. X. Sheng, L. Shen, Z. Qu, M. P. Sulprizio, J. F. Chang, A. A. Bloom, S. Ma, J. Worden, R. J. Parker, H. Boesch, Attribution of the accelerating increase in atmospheric methane during 2010–2018 by inverse analysis of GOSAT observations. Atmos. Chem. Phys. 21, 3643–3666 (2021).
68. T. S. Jayne, P. A. Sanchez, Agricultural productivity must improve in sub-Saharan Africa. Science 372, 1045–1047 (2021).
69. S. Kaza, L. Yao, P. Bhada-Tata, F. Van Woerden, What a waste 2.0: A global snapshot of solid waste management to 2050 (World Bank, 2018); https://datatopics.worldbank.org/what-a-waste/trends_in_solid_waste_management.html.
70. Reuters, Kenya's Mombasa port shows growth, increased efficiency (2015); www.reuters.com/article/ozabs-uk-kenya-ports-idAFKCN0QP0OQ20150820 [accessed 1 July 2021].
71. R. Burnett, H. Chen, M. Szyszkowicz, N. Fann, B. Hubbell, C. A. Pope, J. S. Apte, M. Brauer, A. Cohen, S. Weichenthal, J. Coggins, Q. Di, B. Brunekreef, J. Frostad, S. S. Lim, H. D. Kan, K. D. Walker, G. D. Thurston, R. B. Hayes, C. C. Lim, M. C. Turner, M. Jerrett, D. Krewski, S. M. Gapstur, W. R. Diver, B. Ostro, D. Goldberg, D. L. Crouse, R. V. Martin, P. Peters, L. Pinault, M. Tjepkema, A. Donkelaar, P. J. Villeneuve, A. B. Miller, P. Yin, M. G. Zhou, L. J. Wang, N. A. H. Janssen, M. Marra, R. W. Atkinson, H. Tsang, Q. Thach, J. B. Cannon, R. T. Allen, J. E. Hart, F. Laden, G. Cesaroni, F. Forastiere, G. Weinmayr, A. Jaensch, G. Nagel, H. Concin, J. V. Spadaro, Global estimates of mortality associated with long-term exposure to outdoor fine particulate matter. Proc. Natl. Acad. Sci. U.S.A. 115, 9592–9597 (2018).

72. D. M. Stieb, R. Berjawi, M. Emode, C. Zheng, D. Salama, R. Hocking, N. Lyrette, C. Matz, E. Lavigne, H. H. Shin, Systematic review and meta-analysis of cohort studies of long term outdoor nitrogen dioxide exposure and mortality. PLOS ONE 16, e0246451 (2021).
73. M. Cacciottolo, X. Wang, I. Driscoll, N. Woodward, A. Saffari, J. Reyes, M. L. Serre, W. Vizuete, C. Sioutas, T. E. Morgan, M. Gatz, H. C. Chui, S. A. Shumaker, S. M. Resnick, M. A. Espeland, C. E. Finch, J. C. Chen, Particulate air pollutants, APOE alleles and their contributions to cognitive impairment in older women and to amyloidogenesis in experimental models. Transl. Psychiatry 7, e1022 (2017).
74. S. Y. L. Chua, A. Warwick, T. Peto, K. Balaskas, A. T. Moore, C. Reisman, P. Desai, A. J. Lotery, B. Dhillon, P. T. Khaw, C. G. Owen, A. P. Khawaja, P. J. Foster, P. J. Patel, U. K. B. Eye, C. Vision, Association of ambient air pollution with age-related macular degeneration and retinal thickness in UK Biobank. Br. J. Ophthalmol. bjophthalmol-2020-316218 (2021).
75. Q. Li, D. N. Zheng, Y. Y. Wang, R. Li, H. P. Wu, S. X. Xu, Y. F. Kang, Y. X. Cao, X. J. Chen, Y. M. Zhu, S. G. Xu, Z. J. Chen, P. Liu, J. Qiao, Association between exposure to airborne particulate matter less than 2.5 μm and human fecundity in China. Environ. Int. 146, 106231 (2021).
76. D. E. Schraufnagel, J. R. Balmes, C. T. Cowl, S. De Matteis, S. H. Jung, K. Mortimer, R. Perez-Padilla, M. B. Rice, H. Riojas-Rodriguez, A. Sood, G. D. Thurston, T. To, A. Vanker, D. J. Wuebbles, Air pollution and noncommunicable diseases: A review by the Forum of International Respiratory Societies' Environmental Committee, part 1: The damaging effects of air pollution. Chest 155, 409–416 (2019).
77. X. Zhang, X. Chen, X. B. Zhang, The impact of exposure to air pollution on cognitive performance. Proc. Natl. Acad. Sci. U.S.A. 115, 9193–9197 (2018).
78. R. W. Atkinson, B. K. Butland, H. R. Anderson, R. L. Maynard, Long-term concentrations of nitrogen dioxide and mortality a meta-analysis of cohort studies. Epidemiology 29, 460–472 (2018).
79. A. Vodonos, Y. Abu Awad, J. Schwartz, The concentration-response between long-term PM2.5 exposure and mortality; A meta-regression approach. Environ. Res. 166, 677–689 (2018).
80. J. Lelieveld, C. Barlas, D. Giannadaki, A. Pozzer, Model calculated global, regional and megacity premature mortality due to air pollution. Atmos. Chem. Phys. 13, 7023–7037 (2013).
81. V. A. Southerland, M. Brauer, A. Mohegh, M. S. Hammer, A. van Donkelaar, R. V. Martin, J. S. Apte, S. C. Anenberg, Global urban temporal trends in fine particulate matter (PM2·5) and attributable health burdens: Estimates from global datasets. Lancet Planet. Health 6, e139–e146 (2022).
82. S. C. Anenberg, P. Achakulwisut, M. Brauer, D. Moran, J. S. Apte, D. K. Henze, Particulate matter-attributable mortality and relationships with carbon dioxide in 250 urban areas worldwide. Sci. Rep. 9, 11552 (2019).
83. R. Burnett, C. A. Pope, M. Ezzati, C. Olives, S. S. Lim, S. Mehta, H. H. Shin, G. Singh, B. Hubbell, M. Brauer, H. R. Anderson, K. R. Smith, J. R. Balmes, N. G. Bruce, H. D. Kan, F. Laden, A. Pruss-Ustun, C. T. Michelle, S. M. Gapstur, W. R. Diver, A. Cohen, An integrated risk function for estimating the global burden of disease attributable to ambient fine particulate matter exposure. Environ. Health Persp. 122, 397–403 (2014).

84. E. A. Marais, R. F. Silvern, A. Vodonos, E. Dupin, A. S. Bockarie, L. J. Mickley, J. Schwartz, Air quality and health impact of future fossil fuel use for electricity generation and transport in Africa. Environ. Sci. Technol. 53, 13524–13534 (2019).
85. M. A. Ullah, A. Moin, Y. Araf, A. R. Bhuiyan, M. D. Griffiths, D. Gozal, Potential effects of the COVID-19 pandemic on future birth rate. Front. Public Health 8, 578438 (2020).
86. A. Aassve, N. Cavalli, L. Mencarini, S. Plach, M. L. Bacci, The COVID-19 pandemic and human fertility. Science 369, 370–371 (2020).
87. R. J. van der A, D. H. M. U. Peters, H. Eskes, K. F. Boersma, M. Van Roozendael, I. De Smedt, H. M. Kelder, Detection of the trend and seasonal variation in tropospheric NO2 over China. J. Geophys. Res. Atmos. 111, D12317 (2006).
88. V. Shah, D. J. Jacob, K. Li, R. F. Silvern, S. X. Zhai, M. Y. Liu, J. T. Lin, Q. Zhang, Effect of changing NOx lifetime on the seasonality and long-term trends of satellite-observed tropospheric NO2 columns over China. Atmos. Chem. Phys. 20, 1483–1495 (2020).
89. C. Liousse, E. Assamoi, P. Criqui, C. Granier, R. Rosset, Explosive growth in African combustion emissions from 2005 to 2030. Environ. Res. Lett. 9, 035003 (2014).
90. P. M. Barbosa, D. Stroppiana, J. M. Gregoire, J. M. C. Pereira, An assessment of vegetation fire in Africa (1981–1991): Burned areas, burned biomass, and atmospheric emissions. Global Biogeochem. Cy. 13, 933–950 (1999).
91. K. P. Vadrevu, K. Lasko, L. Giglio, C. Justice, Vegetation fires, absorbing aerosols and smoke plume characteristics in diverse biomass burning regions of Asia. Environ. Res. Lett. 10, 105003 (2015).
92. L. Giglio, J. T. Randerson, G. R. van der Werf, Analysis of daily, monthly, and annual burned area using the fourth-generation global fire emissions database (GFED4). J. Geophys. Res. Biogeo. 118, 317–328 (2013).
93. P. K. Sen, Estimates of the regression coefficient based on Kendall's Tau. J. Am.Stat. Assoc. 63, 1379–1389 (1968).
94. H. Theil, A rank-invariant method of linear and polynomial regression analysis. Proc. K. Ned. Akad. Wet. Series A, 53, 386–392 (1950).
95. R. M. Hoesly, S. J. Smith, L. Y. Feng, Z. Klimont, G. Janssens-Maenhout, T. Pitkanen, J. J. Seibert, L. Vu, R. J. Andres, R. M. Bolt, T. C. Bond, L. Dawidowski, N. Kholod, J. Kurokawa, M. Li, L. Liu, Z. F. Lu, M. C. P. Moura, P. R. O'Rourke, Q. Zhang, Historical (1750–2014) anthropogenic emissions of reactive gases and aerosols from the Community Emissions Data System (CEDS). Geosci. Model Dev. 11, 369–408 (2018).
96. E. E. McDuffie, S. J. Smith, P. O'Rourke, K. Tibrewal, C. Venkataraman, E. A. Marais, B. Zheng, M. Crippa, M. Brauer, R. V. Martin, A global anthropogenic emission inventory of atmospheric pollutants from sector- and fuel-specific sources (1970-2017): An application of the Community Emissions Data System (CEDS). Earth Syst. Sci. Data 12, 3413–3442 (2020).
97. Global Burden of Disease Collaborative Network, GBD 2019 Under-5 Mortality and Adult Mortality 1950–2019. Seattle, United States of America: Institute for Health Metrics and Evaluation (IHME) (2020).
98. World Bank, Population ages (2021); https://data.worldbank.org/topic/health?view=chart [accessed 24 August 2021].
99. P. Zoogman, D. J. Jacob, K. Chance, L. Zhang, P. Le Sager, A. M. Fiore, A. Eldering, X. Liu, V. Natraj, S. S. Kulawik, Ozone air quality measurement requirements for a geostationary satellite mission. Atmos. Environ. 45, 7143–7150 (2011).

100. R. V. Martin, A. M. Fiore, A. Van Donkelaar, Space-based diagnosis of surface ozone sensitivity to anthropogenic emissions. Geophys. Res. Lett. 31, L06120 (2004).
101. B. N. Duncan, Y. Yoshida, J. R. Olson, S. Sillman, R. V. Martin, L. Lamsal, Y. T. Hu, K. E. Pickering, C. Retscher, D. J. Allen, J. H. Crawford, Application of OMI observations to a space-based indicator of NOx and VOC controls on surface ozone formation. Atmos. Environ. 44, 2213–2223 (2010).
102. X. M. Jin, A. M. Fiore, L. T. Murray, L. C. Valin, L. N. Lamsal, B. Duncan, K. Folkert Boersma, I. De Smedt, G. G. Abad, K. Chance, G. S. Tonnesen, Evaluating a space-based indicator of surface ozone-NOx-VOC sensitivity over midlatitude source regions and application to decadal trends. J. Geophys. Res. Atmos. 122, 10439–10461 (2017).
103. A. H. Souri, Y. Choi, W. Jeon, J. H. Woo, Q. Zhang, J. Kurokawa, Remote sensing evidence of decadal changes in major tropospheric ozone precursors over East Asia. J. Geophys. Res. Atmos. 122, 2474–2492 (2017).
104. A. H. Souri, C. R. Nowlan, G. M. Wolfe, L. N. Lamsal, C. E. C. Miller, G. G. Abad, S. J. Janz, A. Fried, D. R. Blake, A. J. Weinheimer, G. S. Diskin, X. Liu, K. Chance, Revisiting the effectiveness of HCHO/NO2 ratios for inferring ozone sensitivity to its precursors using high resolution airborne remote sensing observations in a high ozone episode during the KORUS-AQ campaign. Atmos. Environ. 224, 117341 (2020).
105. J. R. Schroeder, J. H. Crawford, A. Fried, J. Walega, A. Weinheimer, A. Wisthaler, M. Muller, T. Mikoviny, G. Chen, M. Shook, D. R. Blake, G. S. Tonnesen, New insights into the column CH2O/NO2ratio as an indicator of near-surface ozone sensitivity. J. Geophys. Res. Atmos. 122, 8885–8907 (2017).
